# Interleukin 35 Delays Hindlimb Ischemia-Induced Angiogenesis Through Regulating ROS-Extracellular Matrix but Spares Later Regenerative Angiogenesis

**DOI:** 10.3389/fimmu.2020.595813

**Published:** 2020-10-14

**Authors:** Hangfei Fu, Yu Sun, Ying Shao, Jason Saredy, Ramon Cueto, Lu Liu, Charles Drummer, Candice Johnson, Keman Xu, Yifan Lu, Xinyuan Li, Shu Meng, Eric R. Xue, Judy Tan, Nirag C. Jhala, Daohai Yu, Yan Zhou, Kayla J. Bayless, Jun Yu, Thomas J. Rogers, Wenhui Hu, Nathaniel W. Snyder, Jianxin Sun, Xuebin Qin, Xiaohua Jiang, Hong Wang, Xiaofeng Yang

**Affiliations:** ^1^Cardiovascular Research Center, Lewis Katz School of Medicine at Temple University, Philadelphia, PA, United States; ^2^Centers for Metabolic Disease Research, Cardiovascular Research, Thrombosis Research, Departments of Pharmacology, Microbiology and Immunology, Lewis Katz School of Medicine at Temple University, Philadelphia, PA, United States; ^3^Center for Cardiovascular Regeneration, Department of Cardiovascular Sciences, Houston Methodist Research Institute, Houston, TX, United States; ^4^Department of Pathology & Laboratory Medicine Lewis Katz School of Medicine at Temple University, Philadelphia, PA, United States; ^5^Department of Clinical Sciences, Lewis Katz School of Medicine at Temple University, Philadelphia, PA, United States; ^6^Biostatistics and Bioinformatics Facility, Fox Chase Cancer Center, Temple Health, Philadelphia, PA, United States; ^7^Department of Molecular and Cellular Medicine, Texas A&M University College of Medicine, College Station, TX, United States; ^8^Center for Inflammation, Translational and Clinical Lung Research, Lewis Katz School of Medicine at Temple University, Philadelphia, PA, United States; ^9^Center for Translational Medicine, Department of Medicine, Thomas Jefferson University, Philadelphia, PA, United States; ^10^National Primate Research Center, Tulane University, Covington, LA, United States

**Keywords:** IL-35, angiogenesis, ischemia and hypoxia, endothelial cells, IL-12R*β*2

## Abstract

Interleukin (IL) 35 is a novel immunosuppressive heterodimeric cytokine in IL-12 family. Whether and how IL-35 regulates ischemia-induced angiogenesis in peripheral artery diseases are unrevealed. To fill this important knowledge gap, we used loss-of-function, gain-of-function, omics data analysis, RNA-Seq, *in vivo* and *in vitro* experiments, and we have made the following significant findings: *i*) IL-35 and its receptor subunit IL-12RB2, but not IL-6ST, are induced in the muscle after hindlimb ischemia (HLI); *ii*) HLI-induced angiogenesis is improved in Il12rb2−/− mice, in ApoE−/−/Il12rb2−/− mice compared to WT and ApoE−/− controls, respectively, where hyperlipidemia inhibits angiogenesis *in vivo* and *in vitro; iii*) IL-35 cytokine injection as a gain-of-function approach delays blood perfusion recovery at day 14 after HLI; *iv*) IL-35 spares regenerative angiogenesis at the late phase of HLI recovery after day 14 of HLI; *v*) Transcriptome analysis of endothelial cells (ECs) at 14 days post-HLI reveals a disturbed extracellular matrix re-organization in IL-35-injected mice; *vi*) IL-35 downregulates three reactive oxygen species (ROS) promoters and upregulates one ROS attenuator, which may functionally mediate IL-35 upregulation of anti-angiogenic extracellular matrix proteins in ECs; and *vii*) IL-35 inhibits human microvascular EC migration and tube formation *in vitro* mainly through upregulating anti-angiogenic extracellular matrix-remodeling proteins. These findings provide a novel insight on the future therapeutic potential of IL-35 in suppressing ischemia/inflammation-triggered inflammatory angiogenesis at early phase but sparing regenerative angiogenesis at late phase.

## Introduction

Peripheral arterial disease (PAD) is a vascular pathology where narrowed arteries reduce blood flow to the peripheral extremities and organs such as legs, arms, and head. The most common symptom of PAD is leg pain, particularly when walking. PAD is highly associated with coronary artery disease and stroke (1), which are the leading causes of death worldwide. They are caused by atherosclerosis, where the plaques build-up in the artery wall, a common condition in the aging population. It is estimated that more than 200 million people have PAD worldwide (1). Although the current gold standard treatment for advanced PAD patients is surgical revascularization, there are no effective therapies for many patients with advanced conditions who are not candidates for surgery (2). Also, better recovery from surgical revascularization largely relies on angiogenesis in adjacent ischemic tissue. Thus, novel therapies such as pro-angiogenic therapy to improve neovascularization are urgently desired.

Since Dr. Vignali’s team reported the contribution of novel cytokine interleukin-35 (IL-35) to CD4^+^Foxp3^+^ regulatory cell (Treg) function in 2007 (3), significant progress has been made with 629 publications on PubMed. We reported that IL-35 is a new responsive heterodimeric anti-inflammatory cytokine (4). IL-35 belongs to the IL-12 cytokine family and is potent in inhibiting various inflammatory diseases including lipopolysaccharide (LPS)-induced lung inflammation, human aortic endothelial cell (HAEC) activation (5), atherogenic lipids lysophosphatidylcholine (LPC) (6–8) -induced HAEC activation and atherosclerosis (9) but sparing innate immune memory (trained immunity) in HAEC (8, 10–12) as we reported. IL-35 *α*-chain p35 (encoded by IL12A) is shared with IL-12; and *β*-chain EBI3 (encoded by Epstein–Barr virus-induced 3, EBI3) is shared with IL-27 and IL-39. IL-35 receptor (IL35R) subunit IL-12R*β*2 (encoded by IL12RB2) is shared with IL-12 receptor; and IL35R subunit IL-6 signal transducer (IL6ST, gp130) is shared with IL-27 and IL-39 receptors. In addition, EBI3 receptor IL6ST is also shared with IL-6 superfamily, where the IL-12 family belongs. IL-35 signaling is unconventional because it has multiple forms of receptors and presents cell-specificity, as we reviewed (13). It can bind not only to heterodimeric receptor IL-6ST-IL-12R*β*2, but also two homodimers such as: i) IL-6ST-IL-6ST, and ii) IL-12R*β*2-IL-12R*β*2, which then activates signal transducer and activator of transcription 1 (STAT1) and STAT4 in Treg (13). The same study also shows that maximal anti-inflammatory function requires the heterodimeric receptor (5). However, the following study shows that in regulatory B cells (Breg), IL-35 signals through a heterodimer of IL-12R*β*2 and IL-27R*α* and activates STAT1 and STAT3 (14). Unlike other members in the IL-12 family that are secreted mainly by activated antigen-presenting cells (dendritic cells, monocyte/macrophages, and B cells), IL-35 is predominantly secreted by Treg (15) and Breg. To a less extent, it has been predicted to be secreted by other cell types including endothelial cells (ECs) (4).

Angiogenesis is the process of new capillary formation from pre-existing vessels. Inflammation, angiogenesis, and extracellular matrix (ECM) remodeling are often coupled in pathological conditions (16). Inflammation drives angiogenesis by direct and indirect mechanisms, promoting EC proliferation, migration, and vessel sprouting, but also by mediating extracellular matrix remodeling and release of sequestered growth factors, and recruitment of proangiogenic leukocyte subsets (17). In chronic inflammation, the formation of new blood vessels results in an increased endothelial surface area, which further facilitates inflammatory cell migration (18) into the inflamed tissue through vasodilatation and increased endothelial cell permeability (19). Although ECs are the most important cell types (20) in the angiogenic process, immune cell infiltration is critical for angiogenesis in the early stage of ischemia; although sustained inflammation can also lead to delayed angiogenesis (21) or undesired vessel overgrowth (22). Hindlimb ischemia represents a complex model with ischemia-induced necrosis and inflammation, ischemia-induced angiogenesis, arteriogenesis and skeletal muscle regeneration (23). In comparison, scarless wound healing in fetuses has been associated with a short-lived and less robust inflammatory response (21). Thus, anti-inflammatory cytokines are key regulators in balancing the local inflammation and promote functional neovascularization. IL-35 is expressed in many tumor cell lines, and promotes tumor angiogenesis and progression (24–26). However, it has also been shown that IL-35 over-expression increases apoptosis sensitivity and inhibits cell proliferation in human cancer cells (27). Besides, IL-35 inhibits angiogenesis in rheumatoid arthritis, where one study showed that IL-35 can directly inhibit human umbilical vein endothelial cells (HUVECs) angiogenic activities (28). However, the roles of IL-35 in angiogenesis in ischemic vascular diseases are unrevealed.

Mechanistically, angiogenesis involves several biological events, including extracellular matrix (ECM) degradation, pericyte detachment, EC proliferation, migration, and sprouting, the fusion of tip cells, lumen formation, pericyte reattachment, ECM redeposition, and vascular remission and stabilization. Each event requires a balance between pro-angiogenic and anti-angiogenic factors, which is by large mediated by ECM proteins and proteases. Integrins are transmembrane proteins that bind to many types of ECM proteins. They are required for focal adhesion (cell-ECM adhesion) formation that mechanically connects intracellular actin bundles and the extracellular substrate (29). Focal adhesion also bi-directionally transmits molecular signals, and is essential for a variety of cellular activities including cell adhesion, migration, differentiation, and survival (30). In mammals, ECM is a highly dynamic structure composed of ~300 main proteins, including 43 collagen subunits, 36 proteoglycans, and ~200 glycoproteins (31). It provides not only physical support for vascular integrity but also mediates cellular functions such as proliferation and migration. On the other side, matrix metalloproteinases (MMPs), a disintegrin and metalloproteinases (ADAM) and ADAM with thrombospondin motifs (ADAMTS) are the major proteases that collectively can degrade all known ECM components. Thus, they are critical in regulating ECM remodeling during angiogenesis.

Reactive oxygen species (ROS) play an important role in modulating angiogenesis. Low cellular levels of ROS are usually maintained by systems of antioxidant enzymes and their substrates, such as thioredoxin systems (Trx1 and 2) (32). It has been reported that cardiac-specific Trx2 knockout mice recently demonstrated that suppression of mitochondrial ROS can preserve cardiac function by inhibiting apoptosis signal regulating kinase-1-mediated apoptosis (33). However, mitochondrial oxidants can induce phosphorylation of vascular endothelial growth factor receptor 2 (VEGFR2), resulting in activation of the receptor and subsequent angiogenesis. Similar to those findings, our previous reports showed that *i)* IL-35 inhibits lipopolysaccharide (LPS) and proatherogenic lipids LPC induced HAEC activation (5, 7); *ii)* IL-35 suppresses vascular inflammation and atherosclerosis *via* inhibiting mitochondrial ROS(8, 9, 34–37); *iii)* hypoxia may induce thrombus leukocyte transdifferentiation by upregulating endothelial cell-specific angiogenic markers (38); and *iv)* inhibition of caspase-1/inflammasome activation in EC improves ischemia-triggered angiogenesis (39, 40). However, the question remained whether IL-35 modulates ischemia-triggered angiogenesis potentially *via* a ROS-related mechanism.

In this study, we examined a novel hypothesis that IL-35 modulates ischemia-induced angiogenesis through activating integrins and regulating ECM macromolecules. We further hypothesized that IL-35 may dysregulate the core complex of EC-specific adhesion junction (AJ, cell–cell adhesion), which is composed of vascular endothelial cadherin (VE-cadherin), p120-catenin, *β*-catenin or plakoglobin (also known as *γ*-catenin), and *α*-catenin (actin-binding protein). This cadherin-based AJ regulates the intracellular actin-myosin network and is essential for vascular integrity (41, 42). In our study, we used both gain-of-function (IL-35 recombinant protein injection) and loss-of-function (Il12rb2−/−) mouse models to examine the role of IL-35 in ischemia/hypoxia-induced angiogenesis. Protein array and transcriptome analysis were used to explore the underlying molecular mechanisms. Our results have demonstrated for the first time that IL-35 induces ECM remodeling proteins, which leads to the delayed EC migration and tube formation *in vitro*, and the early phase of HLI-induced angiogenesis *in vivo* but spare regenerative angiogenesis at late phase. These findings provide a novel insight on the future therapeutic potential of IL-35 in suppressing ischemia/inflammation-triggered angiogenesis but sparing regenerative angiogenesis.

## Materials and Methods

### Reagents and Antibodies

Recombinant human vascular endothelial growth factor VEGF165 (PeproTech, #100-20), and basic fibroblast growth factor (FGF2) (PeproTech, #100-18B) were reconstituted in 0.1% bovine serum albumin (BSA)/phosphate-buffered saline (PBS). Recombinant mouse IL-35 Fc chimera (Adipogen, #CHI-MF-11135-C025) was reconstituted in sterile PBS. Recombinant human IL-35 Fc chimera (Enzo, #ALX-522-140-C010) was reconstituted in sterile water. Antibodies used against human in western blot: anti-hypoxia-inducible factor-1a (HIF-1a) (BD, #610958), anti-PEDF (Santa Cruz BioTechnology, #sc-390172), anti-*β*-Actin antibody (Sigma #A5441). Antibodies against mouse used in flow cytometry: anti-CD45.1 APC-Cy7 (BioLegend, #110716), anti-CD45 allophycocyanin-cyanine dye 7 (APC-Cy7) (BioLegend, #103116), anti-CD45.2 R-phycoerythrin-Cy7 (PE-Cy7) (eBioscience, #25-0454-82), anti-CD11b BV421 (BD, #562605), anti-CD31 Brilliant™ Violet 605 (BV605) (BioLegend, #102427), anti-CD31 PE-Cy7, anti-CD144 APC (Thermo Fisher, #17-1441-80). Viability dyes used in flow cytometry: live/dead fixable aqua (ThermoFisher, #L34965), 7-aminoactinomycin D (7-AAD) viability solution (eBioscience, #00-6993-50). Antibodies against mouse used in immunofluorescent staining: anti-CD31 (Abcam, #ab28364), anti-CD45 (BioLegend, #103101), anti-vascular endothelial (VE)-cadherin (R&D, #AF1002-SP), anti-NG2 NG2 chondroitin sulfate proteoglycan (Millipore, #ab5320).

### Cell Lines and Cell Culture

Human cardiac microvascular endothelial cells (HMVECs, Lonza, #CC-7030) were cultured in microvascular endothelial cell growth medium-2 (EGM-2 MV) (Lonza, #CC-3202). Human umbilical vein endothelial cells (HUVECs) were cultured in M199 medium (HyClone) supplemented with 20% fetal bovine serum (FBS; HyClone), endothelial cell growth supplement (ECGS, 50 μg/ml, BD Biosciences), heparin (50 μg/ml) and 1% penicillin, streptomycin, and amphotericin (PSA, Invitrogen). Cells were grown on 0.2% gelatin-coated flasks, plates, or dishes in a humidified incubator at 37°C with 5% CO_2_. All *in vitro* assays used HMVEC (purchased at P4, used at P8-9), which was justified to be more pathophysiological relevant angiogenic cell type compared to HUVEC. In 3D sprouting assay, where HMVEC had lower efficiencies in bead-coating than HUVEC, earlier passages of endothelial cell were required for the sprouting phenotype; here HUVECs at P5 were used.

### Fluorescence-Activated Cell Sorting and RNA Isolation for RNA-Seq

To obtain CD45^−^CD31^+^ and CD45^+^CD31^+^ cells from mouse hindlimb, skeletal muscle was dissociated into single-cell suspension through the method reported previously (43). Briefly, hindlimb muscle was dissected, minced, and incubated in muscle dissociation buffer containing collagenase, type 2 (Worthington, #LS004176) and dispase II (Life Technologies, #17105-041) for 1.5 h. After washing with FACS buffer (2% FBS in PBS), cells were stained with surface antibody anti-CD45 (BioLegend, #103116) and anti-CD31 (BioLegend, #102427) for 30 min at room temperature. All cell-sorting experiments were performed using an Aria Cell Sorter (BD Biosciences) in Temple University Lewis Katz School of Medicine Flow Cytometry Core. The CD45^−^CD31^+^ and CD45^+^CD31^+^ cells were sorted directly into TRIzol, and RNA was extracted using miRNeasy Mini kit (Qiagen).

Total RNA libraries were prepared by using Pico Input SMARTer Stranded Total RNA-Seq Kit (Takara). In short, 250 pg–10 ng total RNA from each sample was reverse-transcribed *via* random priming and reverse transcriptase. Full-length cDNA was obtained with SMART (Switching Mechanism At 5′ end of RNA Template) technology. The template-switching reaction keeps the strand orientation of the RNA. The ribosomal cDNA is hybridized to mammalian-specific R-Probes and then cleaved by ZapR. Libraries containing Illumina adapter with TruSeq HT indexes were subsequently pooled and loaded to the Hiseq 2500. Single end reads at 75 bp with 30 million reads per sample were generated for bioinformatic analysis. RNA-seq data was deposited into NCBI-GEO dataset (GSE155012).

### Protein Extraction and Western Blot Analysis

Protein extracts were collected from HMVECs or mouse hindlimb skeletal muscle. Protein concentration was determined by bicinchoninic acid (BCA) assay with BSA standards. Protein was separated on SDS-polyacrylamide gel and transferred onto nitrocellulose membranes. Membranes were blocked with 5% BSA in Tris-buffered saline containing 0.1% Tween 20 [TBST, 50 mM Tris (pH 7.5), 150mM NaCl, and 0.1% Tween 20 (v/v)]. Membranes were incubated with primary antibodies overnight at 4°C, then washed extensively with TBST and incubated with the appropriate horseradish peroxidase-labeled secondary antibodies for 1 h at room temperature. Afterwards, membranes were incubated with enhanced chemiluminescence (ECL) substrate for horseradish peroxidase (Thermo Scientific, #34578) and the ECL intensity was detected by Fujifilm LAS-4000. The expression levels of proteins as indicated by the ECL intensity were measured with ImageJ software. The experiments with human angiogenesis protein array (R&D Systems, #ARY007) were performed following the manufacturer’s protocol.

### Enzyme-Linked Immunosorbent Assay

After euthanizing mice, plasma and skeletal muscle were collected for protein extraction. Less than 50 mg of muscle was added into molar with liquid nitrogen and ground into powder. 400–1,000 µl of lysis buffer (Cloud-Clone, #IS007) was used to extract protein. After sonication at 40 mA for 10 s intermittently and centrifugation at 10,000 g for 5 min at 4°C, supernatants were carefully collected into a new 1.5 ml tube. IL-35 protein expression in mouse plasma and muscle was measured using ELISA kit (Biomatik, #EKU05328) by following its product instruction.

### RNA Isolation and Real-Time Quantitative Reverse Transcription PCR

RNA collected from HMVECs was isolated using the miRNeasy Mini Kit (Qiagen, #217004). RNA from mouse hindlimb skeletal muscle was isolated using RNeasy Fibrous Tissue Mini Kit (Qiagen, #74704). The cDNA was synthesized using High-Capacity cDNA Reverse Transcription Kit (Applied Biosystems, #4368814), and qRT-PCR was performed with iTaq Universal SYBR Green Supermix (Bio-Rad). Samples were amplified by 40 cycles of 5 s at 95°C and 30 s at 60°C. Results were calculated using the ΔΔ C_t_ method relative to the reference control gene of *β*-actin.

For PCR array, HMVECs were treated with 10 ng/ml of rhIL-35 for 6 h, triplicate samples were pooled to collect mRNAs, which were then converted to cDNA and assayed with the RT² First Strand Kit (Qiagen, #330401). The pooled mRNAs were used to screen for 84 EC biology-related gene expression changes following the direction of the Human Endothelial Cell Biology PCR Array (Qiagen, #330231). Data were analyzed with the SABiosciences PCR Array Data Analysis Software.

Human EBI3 (ThermoFisher Scientific, #4331182) and mouse Il12a (Bio-Rad, #10025636) were detected using commercial kits. All other primers were designed and purchased from Integrated DNA Technologies. Primer sequences are listed in **Table 1**.

**Table 1:** Sequences of human and mouse primer pairs.

Species	Gene Name	Forward (from 5′ to 3′)	Reverse (from 5′ to 3′)
Human	ACTB	ACCTTCTACAATGAGCTGCG	CCTGGATAGCAACGTACATGG
Human	IL12A	CTCCAGACCCAGGAATGTTC	ATCTCTTCAGAAGTGCAAGGG
Human	IL6ST	GCAACATTCTTACATTCGGACAG	TCCCACTCACACCTCATTTTC
Human	IL12RB2	ATCTCCCTTTCTGTTTTCCCC	TGAGGGCACACTGACTTTAAG
Mouse	Actin	GGCTGTATTCCCCTCCATCG	CCAGTTGGTAACAATGCCATGT
Mouse	Ebi3	CAAGGAACAGAGCCACAGAG	GGGATACCGAGAAGCATGG
Mouse	Il6st	AGATGAAGGTGGGAAAGATGG	GTTAAAGCAGAACAAGACGCC
Mouse	Il12rb2	GAACGCCTTTTCATTTCCTGG	TGGATGTGAGTTTTGAGAGCG
Mouse	Col1a2	AAGGATACAGTGGATTGCAGG	TCTACCATCTTTGCCAACGG
Mouse	Col11a1	ACAAAACCCCTCGATAGAAGTGA	CTCAGGTGCATACTCATCAATGT
Mouse	Col18a1	CAGACCCTGACAAGTTCCAG	AGCCACTTCCAAAATCTCCAG
Mouse	Adam12	ACAAGTCCAACCTCACCATG	TTCCTTGCCTCTGAAACTCTC
Mouse	Mmp12	CTGCTCCCATGAATGACAGTG	AGTTGCTTCTAGCCCAAAGAAC
Mouse	Mmp14	GGATGGACACAGAGAACTTCG	TTTTGGGCTTATCTGGGACAG
Mouse	Mmp19	GAGCCCAGAGACAAGAGATG	AAGCATAAGTCTTCCCACGAG

### Immunofluorescence Staining

To prepare the frozen sections, collected samples were fixed in 2% paraformaldehyde (PFA) overnight at 4°C, incubated in 15% sucrose in PBS (w/v) for 2 h, and then 30% sucrose overnight at 4°C. Gastrocnemius muscle was cut in the center and embedded with the cut surface facing down in Tissue-Plus O.C.T compound (Fisher Scientific, #23-730-571) and snap frozen on an aluminum block (pre-chilled) in liquid nitrogen. Sections of 10-μm in thickness were cut on Leica CRYOCUT 1800.

For paraffin sections, collected samples were fixed in 4% PFA overnight at 4°C, then transferred into 70% ethanol before embedding. Sections of 5-μm thickness were deparaffinized and treated with a heat-induced antigen retrieval with 1 mM EDTA-NaOH (pH 8.0) solution.

Slides were blocked in PBS with 3% BSA for 1 h at room temperature. Primary antibodies were incubated overnight at 4°C in the blocking buffer. Secondary antibodies were added into the blocking buffer and incubated for 1 h at room temperature. Excess antibodies were washed in PBS with 0.5% Tween 20. Slides were then mounted in VECTASHIELD HardSet Antifade Mounting Medium with 4′,6-diamidino-2-phenylindole (DAPI, Vector, #H-1500-10). Stained sections were imaged by a Leica TCS SP8 confocal microscope using ×10, or ×20 objectives.

### Tube Formation Assay

Reduced growth factor basement membrane matrix (Trevigen, #3433-005-R1) was coated on the 15-well µ-slides (ibidi, #81506) for 30 min. HMVECs were trypsinized, seeded on the coated µ-slides at a density of 10,000 cells/ml in EGM-2 MV medium with or without LPC (30 µM), and incubated for 4–6 h at 37°C. Phase contrast images were taken at ×6.4 magnification with or without Calcein AM (Trevigen, #4892-010-01), a viability fluorescent dye. Images were analyzed using Angiogenesis Analyzer for ImageJ developed by Dr. Gilles Carpentier (http://image.bio.methods.free.fr/ImageJ/?Angiogenesis-Analyzer-for-ImageJ).

### Wound Healing Migration Assay

HMVECs were seeded on 6-well tissue culture plate and cultured until 80% confluence. After starvation overnight in EBM2 plus 0.1% FBS, the monolayer of cells were scratched with 1 ml pipette tip to create an across at the center of the well. Then cells were gently washed twice with medium to remove the detached cells and replenished with fresh starvation medium containing FGF2 (80 ng/ml), with or without IL-35 (40 ng/ml). The wound healing process was monitored for approximately 12 h. Olympus microscopy IX71 was used to image the cells at the first time point T_0_ and the last time point T_12_. For data analysis, ImageJ was used to measure the wound area at T_0_ and T_12._ Migration distance = [Area(T_0_) − Area(T_12_)]/(2 × Length of the field).

### 3D Sprouting Assay

Passage (P) 5 HUVECs were trypsinized and incubated with sterile Cytodex 3 microcarrier beads (GE Healthcare, #17-0485-01) at the ratio of 500 cells per bead in a sterile tube with EGM-2 medium at 37°C for 4 h. During the 4 h, the mix was gently shaken every 20 min. Then, around 250 coated beads were seeded in 0.5 ml fibrin gel (2 mg/ml fibrinogen (Sigma, #F-8630), 0.15 U/ml aprotinin (Sigma, #A-1153), and 0.625 U/ml thrombin (Sigma, #T-4648), 100 ng/ml rhVEGF-A165 (Peprotech, #100-20), 30 ng/ml rhFGF-basic (Peprotech, #100-18B), with or without 30 µM LPC (Avanti, #855675P) in a 24-well plate. After seeding the beads, 1 ml EGM-2 with 20,000 fibroblasts was added dropwise. Culture medium was changed every other day, and beads sprouts were counted at day 10.

### Animals

All mice used were on a C57BL/6 background. Except **Supplementary Figure 2**, in all the other animal experiments, male mice were used. Apolipoprotein E deficient (ApoE−/−) mice (strain name: B6.129P2-Apoetm1Unc/J), Il12rb2^−/−^ mice (strain name: B6.129S1-Il12rb2tm1Jm/J) and wild type (WT) mice (strain name: C57BL/6J) were purchased from the Jackson Laboratory (Bar Harbor, ME). All mice were weaned at 3 weeks of age and maintained on chow diet. In the Matrigel plug assay experiment, severe combined immunodeficiency (SCID) (NOD.Cg-Prkdcscid Il2rgtm1Wjl/SzJ) male mice at 12-week old were used and the experiment was performed with collaborator Dr. Shu Meng from Houston Methodist Hospital; female mice did not response to angiogenic stimuli very well, thus not ideal for this experiment model. In bone marrow transplantation experiment, CD45.1 WT (strain name: B6.SJL-Ptprca Pepcb/BoyJ) mice were used as the recipient and enhanced green fluorescent protein (EGFP) (strain name: C57BL/6-Tg (CAG-EGFP)131Osb/LeySopJ) male mice were used as the donor. In cytokine injection therapy experiment, both male and female mice were used. All animal experiments were performed in accordance with the Institutional Animal Care and Use Committee (IACUC) Guidelines and Authorization for the use of Laboratory Animals and were approved by the IACUC of Temple University Lewis Katz School of Medicine and Houston Methodist Hospital.

### Mouse Genotype

Mouse genotypes were confirmed with PCR followed by agarose gel separation. Extracta DNA Prep for PCR (Quanta, #95091) was used to extract DNA from mouse toe. Briefly, mouse tissue was digested with 50 μl of extraction reagent at 98°C for 30 min, and then added 50 µl of stabilization buffer and stored at 4°C.

IL-12RB2 genes were amplified with primers for the mutant gene (5′-CACGGGTAGCCAACGCTATGTC-3′ and 5′-GCCCTGAATGAACTGCAGGCG-3′) and the WT gene (5′-GTGTGCAAGCTTGGCACTGTGACCGTCCAG-3′ and 5′-GTTTAGCTTGCAG ACAAACAAGGTCATACC-3′). The PCR cycle for IL-12RB2 was 94°C for 3 min, 35 cycles of 94°C for 30 s, 72°C for 1 min, 66.8°C for 1 min, and 72°C for 2 min.

ApoE genes were amplified with 3 primers (5′-GCCTAGCCGAGGGAGAGC CG-3′, 5′-TGTGACTTGGGAGCTCTGCAGC-3′, and 5′-GCCGCCCCGACTGCATCT-3′). And the PCR cycle for ApoE was 3 cycles of 94°C for 5 min, 60°C for 30 s, 72°C for 30 s, and 30 cycles of 94°C for 30 s, 60°C for 30 s, 30°C for 20 s, and 72°C for 7 min.

The PCR product was then mixed with DNA loading dye and separated by gel electrophoresis with a 1.5% agarose gel. The DNA sizes for IL-12RB2 are 265 bp (WT) and 500 bp (mutant), and that of ApoE are 155 bp (WT) and 245 bp (mutant).

### Hindlimb Ischemia Model

Age-matched 10–12-week-old male or female mice were used in HLI model as reported previously (44). Under anesthesia, an incision was made in the skin at the mid-portion of the left hindlimb. The femoral artery was then dissected free from the nerves and the proximal and distal loci of the femoral artery were ligated and cut, as well as its side branches. Hindlimb blood flow was measured on postoperative days 0, 3, 7, 14, 21, and 28 using a laser Doppler imaging (LDI) blood flow analyzer (moorLDI2-IR, Moor Instrument). The penetration depth is ~5 mm when the Infra-Red laser of 785 nm is used (45). Blood flow was quantitatively assessed by the ratio of mean flow signals of the left (ischemic) to the right (non-ischemic) plantar. Mice with blood flow ratio higher than 0.2 at postoperative day 0 were excluded to ensure the success of the ligation procedure, which were correlated well with that reported (46). 80–90% blood loss is typical of what we see after a femoral artery ligation procedure, which is also consistent with the application note on HLI by Moor Instrument. Calf muscles including gastrocnemius and soleus were used for most molecular experiments except flow cytometry. Due to the limited alive cell after single cell suspension, the whole limb muscle was dissected for flow cytometry.

### Matrigel Plug Assay

Matrigel (Corning, #354234) was thawed at 4°C overnight before the experiment. SCID male mice were anesthetized and injected with Matrigel subcutaneously in the shaved abdomen region of the mice (two injections in each mouse). Each Matrigel injection contains 400 µl total volume (300 µl Matrigel + 100 µl other reagents with the final concentrations of 30 U/ml of heparin, 40 ng/ml of FGF2, and with or without 40 ng/ml of IL-35). Matrigel plugs were removed after 5 days for molecular studies.

### Cytokine Injection

Recombinant mouse IL-35 was administered intramuscularly (i.m.) (0.3 µg/gastrocnemius (GC) muscle in 20 µl PBS) at the time of HLI procedure, and three times a week afterwards until the end of observation. The non-surgery limb and PBS control group limb were injected with 20 µl PBS as the internal and experimental control, respectively. The used dose was based on similar literatures using cytokine injection in mouse muscle (47).

### Bone Marrow Transplantation

CD45.1 WT male mice at 5-week old were irradiated with a single dose of 8 min and 29 s in RS2000 X-ray irradiator from Radsource. Then, bone marrow cells were collected from EGFP donor mouse femurs and filtered through a 70 μM cell strainer. Each irradiated CD45.1 mouse was injected by retro-orbital with 5 × 10^6^ donor bone marrow cells. To assess the irradiation efficacy, control mice were irradiated but did not receive donor bone marrow cells. After 6 weeks, all recipient mice survived, while the control mice were dead. Peripheral blood was assessed with flow cytometry, and more than 90% of mononuclear cells were EGFP^+^. The chimeric mice were then performed with HLI surgery and followed with IL-35 or PBS injection.

### Statistical Methods

Data were expressed as the mean ± standard error of the mean (SEM) throughout the manuscript. For comparisons between two groups, the two-tailed Student t-test was used for evaluation. For comparisons across multiple groups, one-way ANOVA with Bonferroni post-test adjustment was used. For the blood perfusion ratio, since the longitudinal data were collected over a 4-week period on mice for the various experimental groups [*e.g.*, 2 strains (WT *vs.* Il12rb2) × 2 treatments (PBS or IL35)] on both sexes, we employed the mixed-effects linear model approach to explore several potential effects of interest, including the gene effect, treatment effect, sex effect, and time effect. Estimation and testing of such effects between various groups of interest were performed using the least squares means method with the Tukey–Kramer adjustments made for multiple comparisons in the mixed-effects regression models while taking into account of the fact that multiple data points were collected on each mouse over time. Variability of the blood perfusion ratio as well as its correlation across different days were allowed to vary by the combinations of time points and groups that were being examined, which is a far better model than that being afforded by a common ANOVA model. Interaction between time and various factors of interest (*e.g.*, strain, treatment, and sex) was always considered in the regression model. Data shown are representative of two to three independent experiments, including analysis from wound healing assay, immunofluorescent staining, flow cytometry, and western blot. *, *p* < 0.05; **, *p* < 0.01; ***, *p* < 0.001; ****, *p* < 0.0001.

## Results

### IL-35 and Its Receptor Subunit IL-12RB2, but Not IL-6ST, Are Induced in the Ischemic Muscle After Hind Limb Ischemia

**Figure 1:**
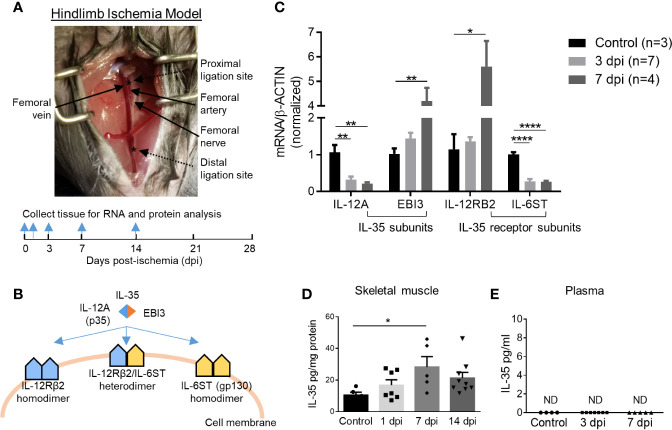
IL-35 and its receptor subunit IL-12RB2, but not IL-6ST, are induced in the ischemic muscle after hindlimb ischemia (HLI). Calf muscle including gastrocnemius and soleus was used in **(C, D)**. **(A)** Schematics of HLI surgery and the time points of tissue collection. **(B)** Schematics of IL-35 signaling complex. **(C)** Normalized gene expression of IL-35 signaling complex in ischemic muscle at pre-ischemia (control), 3 and 7 days post-ischemia (dpi). **(D, E)** IL-35 protein level in skeletal muscle and mouse plasma at indicated dpi. All animal results were collected from male mice. Data are presented as mean ± SEM. ND, not determined; *p < 0.05; **p < 0.01; ****p < 0.0001.

Previous reports showed that a special population of Treg potentiates muscle repair (48); and IL-35 is predominantly secreted by Treg (3, 13), suggesting a possibility that IL-35 may modulate hind-limb ischemia (HLI) angiogenesis related to muscle repair. We hypothesized that IL-35 signaling is induced in HLI angiogenesis model (**Figure 1A**). At physiological conditions, the gene expressions of IL-35 subunits (IL-12A and EBI3) are very low in the skeletal muscle, and protein levels are undetectable based on the Human Protein Atlas database (http://www.proteinatlas.org), which correlated well with our previous report (4). IL-35 signaling involved three possible formats further described in the following results (**Figure 1B**). To test this hypothesis, we extracted RNAs from ischemic muscle at 0, 3, and 7 days post-ischemia (dpi). The results showed significantly increased expression of IL-35 subunit EBI3 and IL-35R subunit IL-12RB2, but reductions of IL-35 subunit IL-12A and IL-35R subunit IL-6ST (**Figure 1C**). The upregulation of EBI3 and downregulation of IL-12A were consistent with the results of the microarray dataset GSE3313 found in NIH Geo DataSets database (https://www.ncbi.nlm.nih.gov/gds/) (**Supplementary Figure 1A**), where gene expression data were also collected from the ischemic mouse muscle after HLI (49). Because IL-35 subunits are shared with other IL-12 family cytokines and the inconsistent changes of the two subunits induced by ischemia, we performed ELISA to detect the changes of IL-35 protein levels in wild-type (WT) mouse plasma and ischemic muscle after HLI. The results showed that IL-35 was induced in ischemic muscle after HLI and peaked at 7 dpi, which was about threefold higher compared to the basal level in non-surgery mouse muscle (**Figure 1D**). However, its expression level was not detectable in plasma at the basal level or after HLI (**Figure 1E**).

To mimic the ischemic environment *in vivo*, human microvascular endothelial cells (HMVECs) were cultured under 0.2% O_2_, where hypoxia-inducible factor 1 (HIF1A) was significantly upregulated (**Supplementary Figure 1B**). HIF1A upregulation indicated the successful establishment of hypoxic environment. The results showed that 24-h treatment of hypoxia induced the gene expressions of IL-35 cytokine subunits IL-12A and EBI3, decreased that of IL-6ST, while did not change that of IL-12RB2 in HMVECs (**Supplementary Figure 1C**). These results suggested that the induction of IL-35 cytokine and its receptor signaling in ischemic muscle the first 12 days after HLI and hypoxic HMVEC was of pathophysiological relevance, and IL-12RB2 was the more responsive IL-35 receptor (IL-35R) subunit than the second IL-35R subunit IL-6ST. These results suggest that IL-12RB2-IL12RB2 homodimer may be responsible for IL-35 signaling in ischemia muscle.

### Hindlimb Ischemia-Induced Angiogenesis Is Improved in IL12RB2−/− Mice, in ApoE−/−/IL12RB2−/− Mice Compared to Wild-Type Controls, and ApoE−/− Controls, Respectively, Where Hyperlipidemia Inhibits Angiogenesis *In Vivo* and *In Vitro*

**Figure 2:**
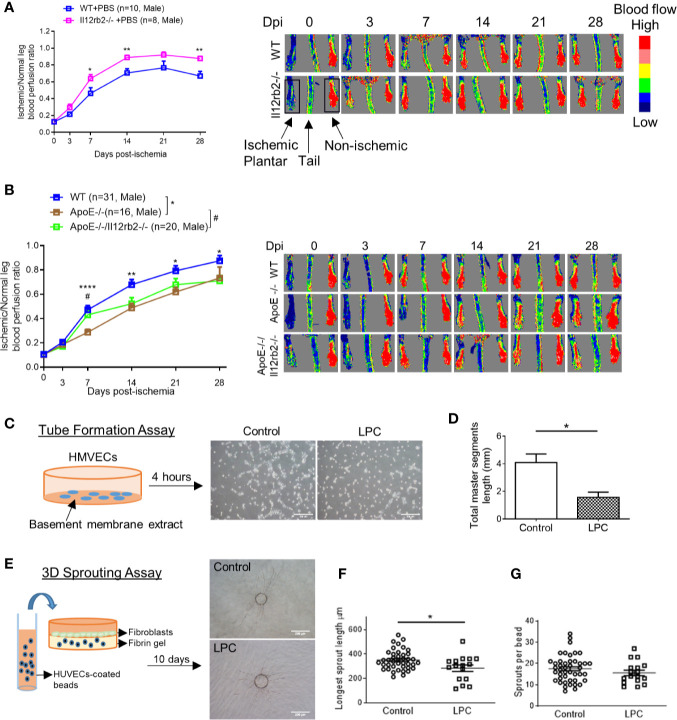
Hindlimb ischemia (HLI)-induced angiogenesis is improved in Il12rb2−/− mice, compared to WT, and in ApoE−/−/Il12rb2−/− mice compared to ApoE−/−, where hyperlipidemia inhibits angiogenesis *in vivo* and *in vitro*. **(A)** Blood perfusion ratio of Il12rb−/− and WT mice in HLI model. Hindlimb blood flow was measured on day post-ischemia (dpi) 0, 3, 7, 14, 21, and 28 with the plantar looking up using a laser Doppler blood flow analyzer. Blood flow was quantitatively assessed by the ratio of mean flow signals of the ischemic to the non-ischemic plantar in black rectangular area. **(B)** Blood perfusion ratio of ApoE−/−/Il12rb2−/−, ApoE−/−, and WT mice in HLI model. **(C)** Experimental schematics and representative images of human microvascular endothelial cell (HMVEC) tube formation. Cells were seeded on Matrigel for 4 h with or without 30 µM of LPC treatment. **(D)** Total master segment length was measured with an ImageJ package of Angiogenesis Analyzer. **(E)** Experimental schematics and representative images of 3D sprouting assay. Human umbilical vein endothelial cell (HUVEC)-coated beads were embedded in fibrin gel for 10 days with or without 30 µM of LPC treatment. **(F)** Statistics of longest sprout length. **(G)** Statistics of sprouts per bead. Sprouts per bead and longest sprout length were manually measured blinded to the treatment. Scale bars are 200 µm in **(C**, **E)**. All animal results were collected from male mice. Data are presented as mean ± SEM. *p < 0.05; **p < 0.01; ****p < 0.0001; ^#^P < 0.05.

IL-35 receptor has a few functional formats (**Figure 1B**), which include: 1) homodimer of IL-12RB2, 2) homodimer of IL-6ST, 3) heterodimer of IL-12RB2 and IL-6ST, and 4) heterodimer of IL-12RB2, and IL-27RA (13, 14, 50). Previously we reported that IL-35 signal is abolished by either IL-12RB2 or IL-6ST inhibition in ECs (5). In our study, the induction of gene expression of IL-12RB2 and reduction of IL-6ST in the ischemic muscle (**Figure 1C**) suggested that IL-12RB2, potential homodimer, played an important role in regulating IL-35 signaling in HLI model. As we recently reviewed (13), IL-12RB2 is the receptor subunit shared by two IL-12 family cytokines, IL-12 (pro-inflammatory) and IL-35 (anti-inflammatory), while IL-6ST as a receptor subunit is widely shared by IL-6 family cytokines, which predominantly drive pro-inflammatory responses. Also, it has been shown that IL-12RB2 deficiency leads to spontaneous autoimmunity in aged mice (51), which indicates that IL-12RB2 is the anti-inflammatory receptor subunit driving the anti-inflammatory function of IL-35 (14, 52). We hypothesized that IL12RB2 mediates IL-35 inhibition of HLI-triggered angiogenesis. To examine this hypothesis, we performed HLI procedure on IL-12RB2 deficient mice. The result showed for the first time that IL-12RB2**−**/**−** mice have significantly improved blood reperfusion in the ischemic legs compared to those of WT control mice (**Figure 2A**), suggesting that IL-35 inhibits HLI-induced angiogenesis *via* IL-12RB2-dependent manner.

One disadvantage of the HLI mouse model to study angiogenesis is that healthy young mice are always used, which is not comparable to aging diseases like PAD (53). Hyperlipidemia is a common complication in patients with PAD. We (**Figure 2B**) and others (54) have seen that hyperlipidemia dampens angiogenesis *in vivo*. We previously reported that gene expressions of IL-35 subunits and its essential receptor subunit IL-12RB2 were induced in mouse aorta after 3-week high-fat diet in ApoE**−**/**−** mice (9), the impaired angiogenesis may partially act through the induction of IL-35. We hypothesized that IL-35 inhibits HLI-triggered angiogenesis in the presence of hyperlipidemia. Thus, to be more clinically relevant, we also used hyperlipidemia model by crossing Il12rb2**−**/**−** mice with hyperlipidemia-conditioned apolipoprotein E deficient (ApoE**−**/**−**) mice. Consistently, ApoE**−**/**−**/Il12rb2**−**/**−** showed significant improvement of blood reperfusion in the ischemic leg at 7 dpi; however, the overall rescue effect of IL-12RB2 deficiency at other time points was not significant (**Figure 2B**). Of note, the kinetics of the response to HLI in C57 wild-type mice and ApoE**−**/**−** mice appeared different, suggesting that hyperlipidemia stimuli inhibit angiogenesis. To verify the effect of hyperlipidemia, two *in vitro* angiogenesis models including EC tube formation assay and three dimensional (3D) sprouting assay showed that 30 µM lysophosphatidylcholine (34) (LPC, a proatherogenic lipid that mimics hyperlipidemia stimuli on EC *in vivo*) restrained tubular network formation in HMVECs (**Figures 2C, D**) and the sprouting length from the 3D beads (**Figures 2E, F**), but not the number of sprouts per bead in HUVECs (**Figure 2G**). The reason that early passage of HUVEC was used in 3D sprouting assay was because HMVEC do not attach to 3D beads well nor sprout well. Our results were also well correlated with that reported by Dr. Belmadani on IL35 cytokine subunit p35 knockout mice fed with chow diet and high fat diet in 14 dpi (55). Of note, as we reviewed recently, IL35 cytokine subunit p35 is shared with IL12 (13). Taken together, these results suggest that IL-35 inhibits HLI-induced angiogenesis in the day 7 after HLI in the presence of hyperlipidemia.

### IL-35 Cytokine Injection Delays Blood Perfusion Recovery at Day 14 After Hindlimb Ischemia and Increases Gene Expression of IL-12RB2

**Figure 3:**
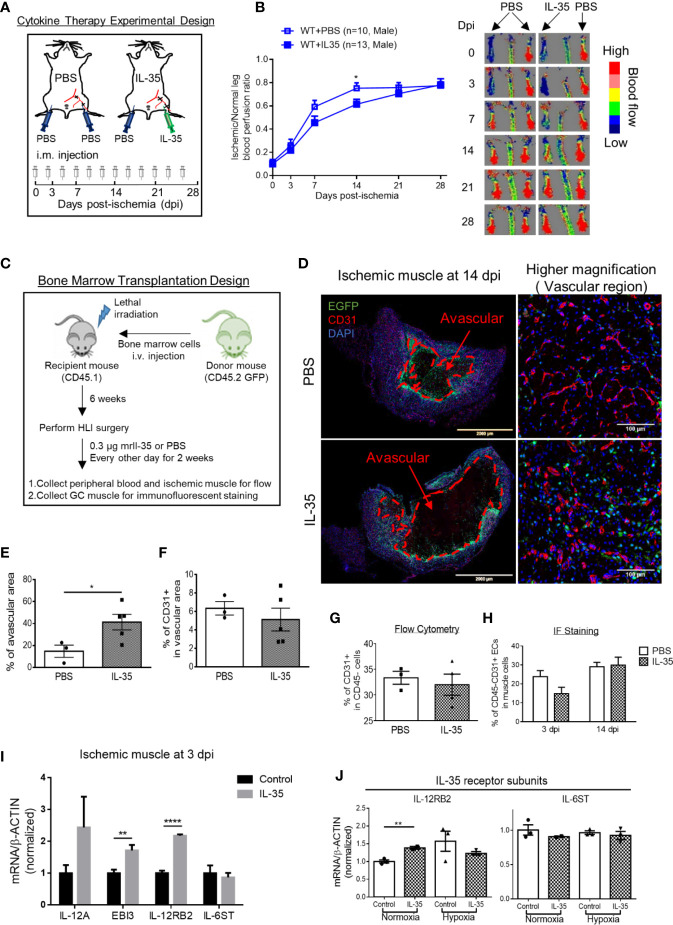
IL-35 cytokine injection delays blood perfusion recovery in the ischemic leg of hindlimb ischemia (HLI) and increases gene expression of IL-12RB2. **(A)** Experimental schematics. IL-35 group mice were intramuscularly (i.m.) administered with IL-35 [0.3 µg/gastrocnemius (GC) muscle in 20 µl PBS] at the time of HLI procedure and three times a week afterwards until the end of observation. The non-surgical limb was also injected with 20 µl PBS as internal control. **(B)** Blood perfusion ratio of IL-35- or PBS-injected WT or Il12rb2−/− mice. **(C)** Bone marrow transplantation experimental schematics. **(D)** Representative images of the whole GC muscle (left panel) and higher magnification of vascular density (right panel) in vascular area of PBS- or IL-35-injected ischemic muscle at 14 dpi. Tissues were stained with anti-CD31 (red) and DAPI. Lines, where blood vessels were rarely visible. The left two images were taken using tile scan in Leica TCS SP8 system and merged to present the whole tissue section. The avascular area was indicated with red dash at 14 dpi. **(E)** Statistics of the percentage of the avascular area EGFP+ indicated infiltrated bone marrow-derived cells. **(F)** Statistics of the percentage of CD31+ cells in the vascular area of the GC muscle at 14 dpi. **(G)** Statistics of percentage of CD31+ cells in CD45− cell population in IL-35 or PBS-injected groups using flow cytometry. **(H)** Percentage of CD45+CD31+ ECs in IL-35 or PBS injected ischemic muscle at 3 and 14 dpi using immunofluorescent staining. **(I)** Normalized gene expression of IL-35 signaling complex in the ischemic muscle at 3 dpi. IL-35 group mice were i.m. injected with IL-35 at the time of HLI surgery. **(J)** Normalized mRNA expression levels of IL-35 receptor subunits after 6 h of 40 ng/ml of IL-35 treatment in HMVECs under normoxia or hypoxia condition. All animal results were collected from male mice. Data are presented as mean ± SEM. *p < 0.05; **p < 0.01; ****p < 0.0001.

As we discussed earlier, IL-12RB2 is the receptor subunit shared by two IL-12 family cytokines, IL-12 and IL-35. Of note, IL-12 has been shown as an anti-angiogenic factor in both tumor (56) and myocardial infarction (57). Thus, we thought that the improvement of angiogenesis in HLI by deleting IL-12RB2 could be merely the effect of blocking IL-12 signaling rather than IL-35. To rule out the possible effect of IL-12 in HLI, we hypothesized that IL-35 cytokine injection inhibits HLI-triggered angiogenesis as the gain-of-function approach. To examine this hypothesis, we use IL-35 injection of recombinant mouse IL-35 protein directly into ischemic muscle after HLI with the method that we reported previously (9). As shown in **Figure 3A**, mice were administered *via* i.m. with IL-35 [0.3 µg/gastrocnemius (GC) muscle in 20 µl PBS] at the time of HLI procedure (47, 58, 59) and three times a week afterward until the end of the observation as we previous reported (60). The results showed that IL-35 injection inhibited blood reperfusion compared to the PBS group, especially at 14 dpi (**Figure 3B**). And this effect of IL-35 at least partially relied on receptor subunit IL-12RB2 (**Supplementary Figure 2A**), where IL-12RB2 deficiency rescued the dampened blood flow recovery by IL-35. We also found that IL-35-IL12RB2 signaling inhibited HLI-induced angiogenesis specially in male mice, and male outperforms female regardless of the treatment or genotype (**Supplementary Figures 2B–F**), which were consistent with previous report (61). Sex differences in vascular physiology and pathophysiology have been well accepted and should be put into consideration for the future studies.

Then, since bone marrow transplantation allows for determining the roles of bone marrow-derived cells *versus* tissue resident cells in modulating HLI and other vascular pathologies as we reported (62), we hypothesized that bone marrow (BM) cells promote HLI-triggered angiogenesis. To test this hypothesis, we performed BM transplantation with enhanced green fluorescence protein (EGFP)-transgenic mouse BM (BM transplantation method, **Figure 3C**). The results showed that IL-35-injected mouse GC muscle after HLI had much bigger avascular areas than that of the controls, judged by significant less CD31^+^ staining and surrounding EGFP^+^ BM-derived cells at 14 dpi (**Figures 3D, E**). However, we did not see significant changes of vascular density at the vascular area, judged by the percentage of CD31^+^ area in the field of view of IL-35-treated HLI mice (**Figure 3F**), suggesting that IL-35 inhibitory effects was focused on HLI-triggered angiogenesis. In addition, the EGFP+ BM-derived cells were in high concentration in vascular areas but not in avascular areas, suggesting that EGFP+ BM-derived cells facilitate angiogenesis in the presence of IL-35. Of note, we did not find the evidence that GFP^+^ BM-derived cells incorporate into CD31^+^ cells (not shown). This result was confirmed by another experiment with muscle samples analyzed by flow cytometry and immunofluorescent (IF) staining (**Figures 3G, H**), though there was a trend of decreasing EC percentage at the earlier time point (3 dpi) of HLI in the IL-35-injected group. To confirm the inhibitory role of IL-35 in angiogenesis, we performed another *in vivo* angiogenesis model, Matrigel plug assay, where IL-35-contained plugs showed significantly less growth of new blood vessel (**Supplementary Figure 3**). These results suggest that IL-35 inhibits HLI-triggered angiogenesis in the HLI-affected area of tissue but not in the HLI-non-affected areas of tissue.

We also found that IL-35 injection upregulated gene expression of IL-12RB2 but not IL-6ST in the ischemic muscle at 3 dpi (**Figure 3I**). The above qRT-PCR assay was performed using the whole muscle extracts, which included many cell types such as immune cells, vascular smooth muscle cells (VSMCs), and ECs. The induction of both IL-35 subunits, IL-12A and EBI3, implicated that exogenous IL-35 could stimulate these immune cells (14, 50) and/or ECs in the ischemic muscle to secrete more IL-35. In our previous findings, IL-35 can directly signal through EC and inhibit LPC- or LPS-induced EC activation (5, 8). Therefore, we treated HMVEC with IL-35 for 6 h under normoxia or hypoxia to examine the direct effect on EC. Consistently, the result showed that gene expression of IL-12RB2, but not IL-6ST, was induced in normoxia, though not in hypoxia (**Figure 3J**).

### IL-35 Spares Regenerative Angiogenesis at the Late Phase of Hindlimb Ischemia Recovery

Our previous report showed that inhibition of HLI-activated danger associated molecular pattern receptors (DAMP-Rs) caspase-1/inflammasome activation in EC improves angiogenesis at 1 dpi, 3 dpi, and 7 dpi but not at the late phase in 10 dpi and 21 dpi (39), suggesting that after 7 dpi, pro-inflammatory mechanisms are decreased and anti-inflammatory mechanisms play more significant roles than pro-inflammatory mechanisms. We found that IL-35 did not significantly inhibit the regenerative angiogenesis in the late phase of hindlimb ischemia-triggered angiogenesis in 21 and 28 dpi (**Figure 3B**), which were well correlated with our other two results that *first*, IL35 cytokine levels were increased at 1, 7, and 14 dpi and were peaked at 7 dpi (**Figure 1D**); *second*, the deficiency of IL12RB2 in ApoE**−**/**−** background did not significantly improve angiogenesis at 21 and 28 dpi (**Figure 2B**); on those two time points the blood perfusion in the deficiency of IL-12RB2 had no differences with that of ApoE**−**/**−** single gene knockout controls. Of note, one of the discrepancies was that IL12RB2**−**/**−** mice have significantly improved blood reperfusion in the ischemic legs at all the time points compared to those of WT control mice (**Figure 2A**), which may result from the fact that IL-12RB2 is the receptor subunit shared by two IL-12 family cytokines, IL-12 and IL-35; and IL-12 may inhibit regenerative angiogenesis. A previous report showed that at 21 dpi, HLI-induced muscle injury is almost fully recovered as judged by the expressions of 18 chemokines and receptors, 23 cytokines and receptors, 26 energy metabolism regulators and histological recovery of muscles (63). The findings suggested that starting from 14 dpi, HLI-triggered muscle injury experiences regenerative angiogenesis and muscle repair, which continues to 28 dpi. Therefore, taken together, our new findings suggest that IL-35 inhibits HLI-triggered inflammatory angiogenesis at the early phase and spare regenerative angiogenesis at the late phase of HLI recovery.

### Transcriptome Analysis of Endothelial Cells at 14 Days Post-Ischemia Reveals a Disturbed Extracellular Matrix Re-Organization in IL-35-Injected Mice

**Figure 4:**
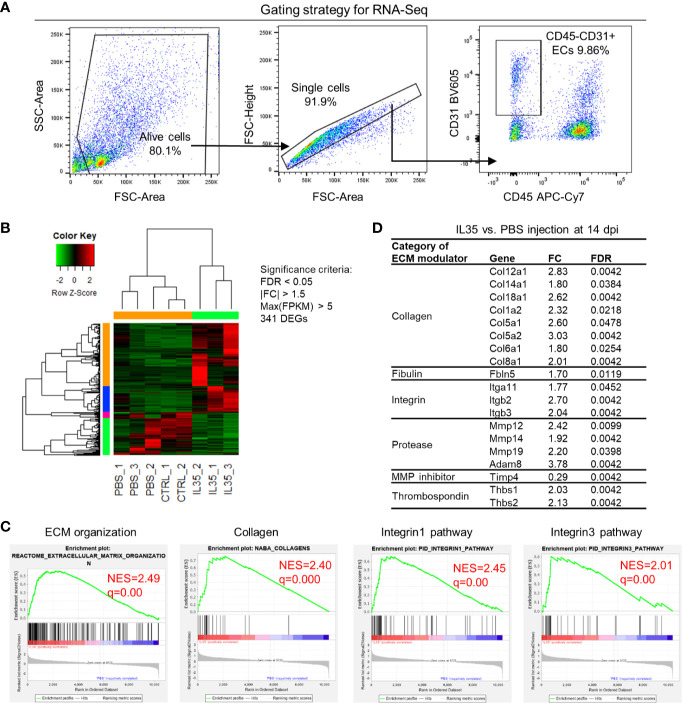
Transcriptome analysis of endothelial cells (ECs) at 14 days post-ischemia (dpi) reveals a disturbed extracellular matrix (ECM) reorganization in the IL-35-injected mice. **(A)** Flow cytometry cell sorting strategy. Hindlimb muscle from control (Ctrl, without surgery) or ischemic mouse (PBS and IL-35 groups) at 14 dpi was dissected and digested for single cell suspension. Cells were stained with the surface markers of CD31 BV605 and CD45 APC-Cy7 and sorted by Aria Cell Sorter (BD Biosciences). 1 × 10^5^ cells of ECs from each sample were collected for RNA extraction and deep-sequencing. **(B)** Heatmap of the differentially expressed genes (DEGs) in ECs of IL-35- or PBS-injected, and control groups. With the criteria of false discovery rate (FDR) < 0.05, |FC| > 1.5, and max (fragments per kilobase of transcript per million mapped reads, FPKM) > 5, we got 341 DEGs. The green-red color key was scaled based on the z-score of FPKM within the row. **(C)** Gene set enrichment analysis (GSEA) of the gene expression changes of ECs in IL-35-injected mouse compared to that of PBS group. Four of the most enriched pathways were presented. **(D)** Representative gene expression changes in different categories of ECM proteins. All results were collected from male mice. n = 3.

We hypothesized that IL-35 regulates angiogenesis *via* modulating the transcriptome of EC in the muscle after HLI. To examine this hypothesis, we specifically isolated CD45^−^CD31^+^ *bona fide* EC from the ischemic muscle (**Figure 4A**) at 0, and 14 dpi using fluorescence-activated cell sorting (FACS). About 0.1 million ECs were collected for RNA extraction and deep-sequencing. A comparison of EC RNA-Seq data (transcriptome) of IL-35 injection and PBS control was made at 14 dpi since our data presented above showed that IL-35 inhibits angiogenesis at 14 dpi (**Figure 3B**). Consistent with the Laser Doppler result that the blood flow of PBS-injected group recovered about 80% of that of non-ischemic leg at 14 dpi, we found this group had a very similar transcriptomic pattern compared to the control group (without HLI surgery). However, the IL-35-injected group showed a quite different gene expression profile. With the significance criteria of false discovery rate (FDR) <0.05, |fold change (FC)| >1.5 and Max [fragments per kilobase of exon model per million reads mapped (FPKM)] >5, there were 341 differentially expressed genes (DEGs) in IL-35-injected ECs (**Figure 4B**). Gene set enrichment analysis (GSEA) (https://www.gsea-msigdb.org/gsea/index.jsp) revealed several significant enriched gene sets regulating ECM remodeling, including molecular pathways of collagen, and integrin *β*1 and *β*3 (**Figure 4C**).

**Figure 5:**
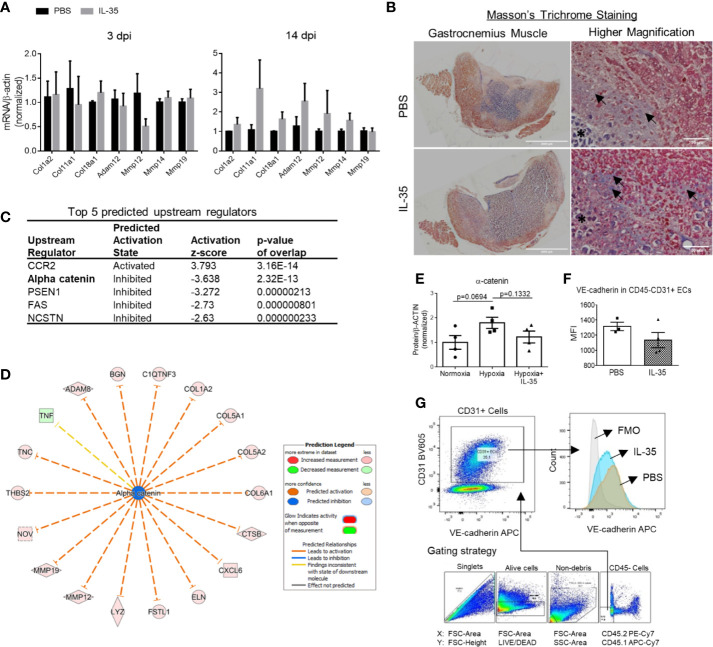
IL-35 induces extracellular matrix genes *in vivo*, possibly *via* impairing the core complex in endothelial cell adhesion junction, (VE-cadherin)–plakoglobin–(*α*-catenin). **(A)** Confirmation of RNA-Seq results by qRT-PCR. Representative genes included collagens and matrix metalloproteinases, which were significantly upregulated in RNA-Seq data. After IL-35 or PBS injection following HLI for 3 or 14 days (same as **Figure 3A**), RNA was extracted from the ischemic muscle. n ≥ 3. **(B)** Representative images of Masson’s trichrome staining of gastrocnemius (GC) muscle (left panel) and higher magnification (right panel) of non-necrotic areas (black arrows) showing the collagen deposition. The stars indicated necrotic tissues with dark purple staining. **(C)** The top five upstream regulators predicted to be significantly activated or inhibited using IPA core analysis. **(D)** The network of *α*-catenin regulating 18 downstream molecules that were significantly changed in IL-35-treated ECs in the ischemic muscle at 14 days post-ischemia (dpi). **(E)** Statistics of *α*-catenin protein level in human microvascular endothelial cells (HMVECs). **(F)**. The gating strategy of mouse EC from ischemic muscle. Digested muscle cells were first gated for singlet with forward scatter (FSC)-Area and FSC-Height, alive cells with FSC-Area and LIVE/DEAD (stains only dead cells), non-debris with FSC-Area and side scatter (SSC)-Area, and then CD45− cells with antibodies of CD45.2 PE-Cy7 and CD45.1 APC-Cy7. **(C)** Density plot with antibodies of CD31 BV605 and VE-cadherin APC. CD31+ ECs were gated from the CD45- cell population. **(G)** Statistics of VE-cadherin median fluorescent intensity (MFI) in IL-35 or PBS-injected groups. All animal results were collected from male mice. Scale bar sized are as indicated in the pictures. Data are presented as mean ± SEM.

This enrichment gene set profile is likely due to the IL-35-upregulated genes in the collagen family (eight genes including types I, V, VI, VIII, XII, XIV, and XVIII), matrix metalloproteinase (MMP) family (three genes including Mmp12, Mmp14, and Mmp19), and integrin molecules (three genes including Itgb2, Itgb3, and Itga11). Representative gene expression changes were listed in **Figure 4D**. Due to the limited cell numbers of CD45^−^CD31^+^ endothelial cells sorted from ischemic muscle at 14 dpi, we did qRT-PCR on RNAs collected from whole ischemic muscles instead to verify the gene expression changes. The tested genes did show an upregulation trend at 14 dpi, but not at 3 dpi (**Figure 5A**). We also saw the increasing trend of collagen deposition in ischemic gastrocnemius (GC) muscle at 14 dpi (**Figure 5B**), which was consistent with the recently reported observations of IL-35 in myocardial infarction (64). Moreover, to consolidate the finding, additional collagen gene expressions were analyzed. The expression changes of eight out of 47 collagen-related genes (**Supplementary Figure 4**) were significantly upregulated in our CD45^−^CD31^+^ mouse endothelial cells from IL-35-treated mice in comparison to that of non-treated control mice (**Figure 4D**), which suggests that IL-35 may increase collagen deposition by upregulating collagen-related gene expression. The proper angiogenic process in which new blood vessels sprout from existing ones largely relies on timely regulation of ECM degradation and synthesis. These over-activated ECM remodeling genes by IL-35 at 14 dpi of HLI indicated the inhibited angiogenesis in the early stage and therefore delayed vascular/tissue repair.

### IL-35 Downregulates Three Reactive Oxygen Species (ROS) Promoters and Upregulates One ROS Attenuator, Which May Functionally Mediate IL-35 Upregulation of Anti-Angiogenic Extracellular Matrix Proteins in ECs

Dr. Kevil’s team reported that sodium nitrite significantly restores ischemic tissue perfusion by 3 dpi, which returned to normal by 7 dpi (65). In addition, Dr. Belmadani’s team reported that nicotinamide adenine dinucleotide phosphate (NADPH) oxidase 4 (Nox4) is significantly increased in ischemic muscle in HLI (55). These reports demonstrated that ROS and RNS play a significant role in modulating the pathophysiological processes of HLI. Recently, we reported that IL-35 inhibits human aortic EC activation *via* suppressing ROS generation (8, 9), mitogen-activated protein kinase pathway (5), epigenetic (9) and gene transcription (8). Of note, our experimental data have demonstrated for the first time that IL-35 not only suppresses inflammation but also inhibits ROS-mediated processes. To further demonstrate that ROS play significant roles in modulating HLI-triggered inflammation and regeneration, we examined the 165 ROS regulators, a comprehensive list, in the GSEA database in the time course, 1, 7, and 14 dpi by using the wild-type male mouse microarray datasets GSE3313 in the NIH GEO database (https://www.ncbi.nlm.nih.gov/gds/) (49). As shown in **Supplementary Figure 5**, the expressions of 116 out of 165 ROS regulators (70.3%) were significantly modulated in the different time courses after HLI, suggesting that ROS regulators play significant roles in modulating inflammatory angiogenesis, which we can classify into two groups for the first time, the early phase inflammatory angiogenesis ROS regulators and the late phase regenerative angiogenesis ROS regulators. It has been reported that hypoxia triggers reactive oxygen species (ROS)-mediated inflammatory cell recruitment, migration and inflammation (66, 67), which may be the dominant feature in the early phase in HLI. Our previous report showed that IL-35 inhibits mitochondrial ROS-mediated HAEC activation and atherosclerosis. In the regenerative phase, as blood supply restores, hypoxia-triggered inflammation is decreased. Tissue remodeling and regeneration become the dominant feature. The mild hypoxia promotes survival and proliferation even in the absence of mitochondrial form of manganese superoxide dismutase (SOD2) (68, 69). As we reviewed recently (13), IL-35 promotes Treg generation, and Treg facilitates tissue remodeling and regeneration (70). Future work is needed to characterize the detailed molecular mechanisms and features of these newly proposed two phases of HLI. Taken together, our results and analysis suggest that oxygen tension and ROS (71) within the muscle may orchestrate the dominant events and transition of the two phases of HLI.

**Figure 6:**
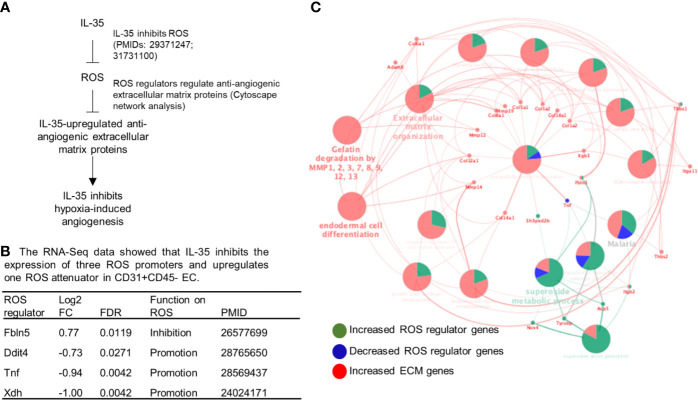
IL-35 inhibits the expression of reactive oxygen species (ROS) regulators including downregulation of ROS promoters such as Ddit4, TNF and Xdh and upregulation of ROS attenuator fibulin-5 (Fbln5), which are connected to downstream extracellular matrix proteins, revealed by Cytoscape analysis. **(A)** A schematic presentation of IL-35 inhibition of reactive oxygen species generation and IL-35 upregulation of anti-angiogenic extracellular matrix proteins. Of note, we have two publications that clearly indicate IL-35 inhibition of ROS generation (the PMIDs included). **(B)** Our RNA-Seq data analysis indicated that IL-35 downregulates three ROS promoter genes including DNA damage inducible transcript 4 (Ddit4, REDD1, HIF-1 responsive RTP801), tumor necrosis factor-a (TNF), and xanthine dehydrogenase (Xdh, XOR) and upregulates ROS inhibitor fibulin-5 (Fbln5) in CD45-CD31+ endothelial cells (EC) treated with IL-35 *versus* PBS-treated controls with the criteria of false discovery rate (FDR) < 0.05. The regulatory functions of these four ROS regulators were reported and the PMIDs were included. Of note, the expression of 165 ROS regulators were examined in the Gene Set Enrichment Analysis (GSEA, https://www.gsea-msigdb.org/gsea/msigdb/cards/GO_REACTIVE_OXYGEN_SPECIES_METABOLIC_PROCESS). **(C)** The network data integration database Cytoscape analysis (https://cytoscape.org/) was used to analyze the network connection between ROS regulators and anti-angiogenic extracellular matrix proteins. The small dots were the ROS regulator genes and extracellular matrix genes that IL-35 regulated. The large dots were extracellular matrix protein pathways. The bright words, but not dim words, indicated the significance of the pathways.

To determine whether ROS inhibition serves as a novel mechanism underlying IL-35 modulation of angiogenesis, we hypothesized that IL-35 inhibition of ROS may mediate IL-35 upregulation of anti-angiogenic extracellular matrix proteins discussed above (**Figure 6A**). To test this hypothesis, we first determined whether IL-35 inhibits some ROS-promoting genes and upregulates ROS-attenuating genes. As shown in **Figure 6B**, the expressions of 165 ROS regulator genes in the GSEA database were examined in the RNA-Seq date of IL-35-treated mouse CD45^−^CD31^+^ ECs in comparison to that of PBS treated mouse ECs. We found that IL-35 significantly downregulates the expressions of three ROS promoters including DNA damage inducible transcript 4 (Ddit4, REDD1, or HIF-1 responsive RTP801), tumor necrosis factor-α (TNF), and xanthine dehydrogenase (Xdh, XOR) and upregulates ROS inhibitor fibulin-5 (Fbln5), which were well correlated with IL-35 suppression of ROS in ECs as we reported (8, 9). We noticed that IL-35 inhibited ROS promoters DDIT4 and XDH were in the inflammatory angiogenesis ROS promoter group (**Supplementary Figure 5**), suggesting that IL-35 inhibits inflammatory angiogenesis ROS promoters at early phase. We also noticed that IL-35 upregulated ROS inhibitor Fbln5 was in the late regenerative angiogenesis ROS regulator group (**Supplementary Figure 5**). Of note, the functions of these four ROS regulators have been reported as the PMIDs shown in **Figure 6B**.

We then hypothesized that these four ROS regulators are functionally connected to IL-35 upregulation of anti-angiogenic extracellular matrix proteins revealed by our RNA-Seq. To test this hypothesis, we used the Cytoscape network integration database. As shown in **Figure 6C**, four ROS regulators (small dots) were functionally connected to extracellular matrix proteins (also small dots) *via* ECM pathway network (large dots). Since we previously demonstrated in the extensive experiments that IL-35 inhibits ROS generation, and that these four ROS regulators have been experimentally verified in the literature (**Figure 6B**), taken together, these results suggest that IL-35 inhibition of ROS generation may mediate IL-35 upregulation of anti-angiogenic ECM.

### IL-35 Inhibits Human Microvascular EC Migration and Tube Formation *In Vitro* Mainly Through Upregulating Anti-Angiogenic Extracellular Matrix-Remodeling Proteins

**Figure 7:**
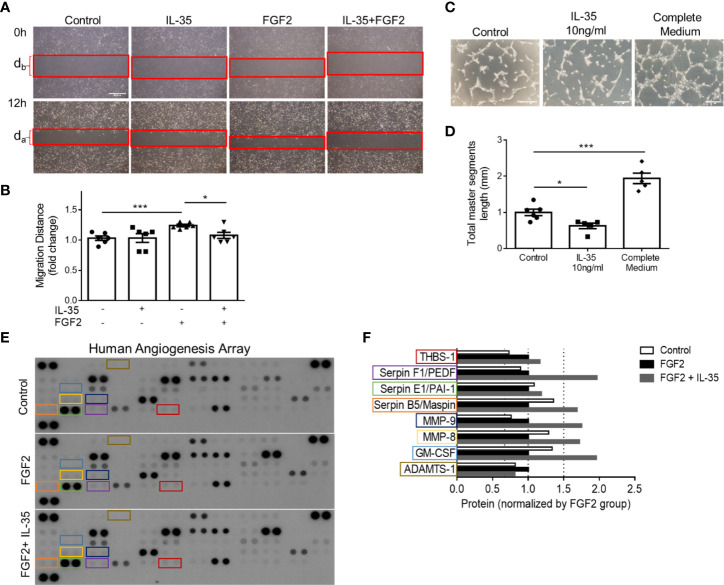
IL-35 inhibits human microvascular endothelial cell (HMVEC) migration and tube formation *in vitro* mainly through upregulating anti-angiogenic extracellular matrix-remodeling proteins. **(A, B)** Representative images and statistics of wound healing assay. Scale bar is 500 µm Migration distance (mm) = (d_b_ − d_a_)/2. After starvation with 0.1% FBS overnight, the monolayers of HMVECs were scratched and treated with/without 40 ng/ml of IL-35, 80 ng/ml of FGF2, or both IL-35 and FGF2 for 12 h. Images were taken at 0 and 12 h. Two individual experiments were combined in **(B)**. **(C, D)** Representative images and statistics of tube formation assay. Scale bar is 200 µm. After starvation with 0.1% FBS overnight, HMVECS were seeded on basement membrane extract (BME) and treated with/without 10 ng/ml of IL-35, or complete culture medium for 5 h. Total master segment length was measured using ImageJ Angiogenesis Analyzer. Two individual experiments were combined in **(D)**. **(E)** Three blots of human angiogenesis protein array. After starvation with 0.1% FBS overnight, HMVECs were treated with/without 80 ng/ml of FGF2, or both 40 ng/ml of IL-35 and FGF2 for 12 h. Proteins were collected and pooled from three biological replicates in each treatment, then performed proteome profile following the instruction of human angiogenesis array kit. One-way ANOVA with Bonferroni post-test adjustment was used based on two technical replicates of each protein. IL-35-changed proteins under FGF2 treatment were color-labeled and framed. **(F)** Normalized expression of IL-35-changed angiogenic proteins compared to FGF2-treated group. Data from **(B, D)** are presented as mean ± SEM. *p < 0.05; ***p < 0.001. Data from **(F)** has no error bar or statistics because each group only has one pooled sample for the protein array.

We hypothesized that IL-35 upregulates anti-angiogenic ECM proteins not only in transcription levels but also in protein expression. To demonstrate the direct function of IL-35 to HMVECs during the angiogenic process, we performed two *in vitro* assays, tube formation and wound healing (**Figures 6A, B**). Under angiogenic stimuli of fibroblast growth factor 2 (FGF2) (72), IL-35 significantly reduced HMVEC migration (**Figures 7A, B**). Though IL-35 did not inhibit HMVEC migration in the basal level, it could significantly impair tube formation. IL-35-retarded EC tube formation and migration has been reported in HUVECs by suppressing Ang2/Tie2 pathway (28); however, we did not observe the transcriptional changes of Ang2/Tie2 in the IL-35-treated HLI-isolated mouse ECs. The results showed that IL-35 inhibited tube formation but not wound healing at the basal levels (**Figures 7C, D**), suggesting that IL-35 inhibition of angiogenesis acts on vessel formation but not on angiogenic cell migration at basal levels. These results were well correlated with our results in **Figure 3E** above that the EGFP^+^ BM-derived cells (including potential angiogenic cells) (73) were in high concentration in vascular areas in the presence of IL-35. To identity the molecular mechanisms underlying IL-35 suppression of tube formation and FGF2-induced EC migration, we performed a human protein array assay, which included 55 angiogenesis-related proteins (**Figure 7E**). Briefly, HMVECs were treated with medium only, 80 ng/ml of FGF2, or both FGF2 and IL-35 (40 ng/ml) for 12 h. Consistent with the mouse EC RNA-Seq result that most significantly enriched pathways in IL-35-injected group were related to ECM organization, this result showed that IL-35 regulated nine angiogenic proteins, and eight of them (89%) were ECM-modulating proteins (**Figure 7F**). Specifically, IL-35 upregulated several anti-angiogenic proteins, including plasminogen activator inhibitor 1 (PAI-1), pigment epithelium-derived factor (PEDF) (confirmed in **Supplementary Figure 6**), Maspin (a tumor suppressor), and thrombospondin 1 (THBS-1, an endogenous inhibitor of angiogenesis). The last three proteins have been shown to inhibit EC migration directly *in vitro* (74–76). In addition, transcriptional changes of the tested genes had similar extent of increase in both mouse ECs isolated from IL-35-injected muscle and in IL-35-treated HMVECs. PAI-1 (encoded by Serpine1) was upregulated by 2.11-fold in mouse ECs and 1.17-fold in HMVECs; PEDF (encoded by Serpinf1) was upregulated by 1.34-fold in mouse ECs and 2.28-fold in HMVECs; and Thbs1 was upregulated by 2.03-fold in mouse ECs and 1.15-fold in HMVECs (HMVEC results from PCR array did not show). Taken together, IL-35 inhibits angiogenesis largely *via* the induction of anti-angiogenic ECM-remodeling regulators in transcription and protein levels *in vitro* and *in vivo* presumably *via* a novel mechanism of IL-35 modulation of ROS (**Figure 6**).

## Discussion

PAD affects approximately 20% of adults over the age of 50 (77). PAD is an aging disease caused by atherosclerosis, where patients often present impaired angiogenic potential after ischemia. Inflammatory regulators are critical for post-ischemic neovascularization. However, sustained inflammation leads to delayed neovascularization (21) or undesired excessive growth of blood vessels (22). HLI model is the main preclinical experimental model for PAD. It has been a powerful model to study post-ischemic revascularization, postnatal arteriogenesis, and angiogenesis (44). However, there are several limitations. *First*, common complications such as atherosclerosis and diabetes are not always considered in the animal model, where in most cases young mice are used for experiments and are not comparable to aging diseases (53). *Second*, surgical ligation in HLI model causes an acute ischemia rather than a chronic process of vascular narrowing seen in patients (77–79). Diabetic mice have been shown to present impaired angiogenesis and recovery from HLI. IL-35 has been reported to inhibit diabetic neuropathic pain (80). Due to the limited scope of this study, we were unable to include diabetic conditions into our consideration (64). Future work is needed to determine the roles of IL-35 in suppressing diabetic inflammation and modulating diabetes-impaired angiogenesis. *Third*, there are variabilities in the angiogenic processes in different mouse strains (78). *Fourth*, we acknowledge that current one stage HLI model (81) would not allow us to examine the regenerative angiogenesis in details. Thus, anti-inflammatory treatment has been presumed a promising therapy to control the local inflammation and promote desired neovascularization. TGF-*β*, IL-10, and newly discovered cytokine IL-35 are three primary anti-inflammatory cytokines secreted by Tregs, which are the critical immunosuppressive cells in our body (13, 26, 82, 83). TGF-*β* is a multifunctional regulator constitutively expressed in many tissues (4), while IL-10 and IL-35 are inflammation-induced anti-inflammatory cytokines (4). This suggests that IL-10 and IL-35 could be better therapeutic targets in the treatment of chronic inflammatory diseases. Emerging studies have shown that IL-35 is a more potent anti-inflammatory cytokine in promoting inducible Treg (50) and Breg (14) than TGF-*β* and IL-10. In addition, human Tregs express and require IL-35 for maximal suppressive function, and activated human Tregs have substantially upregulated gene expression of EBI3 and IL-12A, but not IL-10 or TGF-*β* (84). While TGF-*β* and IL-10 have been extensively studied, the role of IL-35 in angiogenesis has not been thoroughly investigated. It has been shown that IL-35 is expressed in many tumor cell lines compared to normal cell lines and promotes tumor angiogenesis and progression (24, 25). However, one study finds that IL-35 over-expression inhibits tumor angiogenesis through increasing apoptosis sensitivity and inhibition of proliferation in human cancer cells (27). In rheumatoid arthritis (28), it has been demonstrated that IL-35 inhibits angiogenesis. However, the roles of IL-35 in modulating angiogenesis triggered by HLI remained unknown. Our results showed that IL-35 therapy delayed inflammatory angiogenesis phase but did not make a significant difference at regenerative angiogenesis phase, suggesting that IL-35 therapy made a catch-up in facilitating the regenerative phase. Future work is required to determine the molecular and cellular mechanisms underlying the catch-up and facilitation, which were well correlated with IL-35 promotion of macrophage survival and improvement of wound healing after myocardial infarction (85). In addition, differences between the IL-35 roles in angiogenesis in HLI and myocardial infarction may also result from potential functions of cardiokines (86) and myokines (87).

To fill this important knowledge gap, we examined this issue by using loss-of-function, gain-of-function, omics data analysis, RNA-Seq, *in vivo* and *in vitro* experiments, and we have made the following significant findings: *i)* IL-35 and its receptor subunit IL-12RB2, but not IL-6ST, are induced in the ischemic muscle after HLI; *ii)* HLI-induced angiogenesis is improved in IL12RB2−/− mice, in ApoE−/−/IL12RB2−/− mice compared to WT and ApoE−/− controls, respectively, where hyperlipidemia inhibits angiogenesis *in vivo and in vitro; iii)* IL-35 cytokine injection delays blood perfusion recovery at day 14 after HLI; *iv)* IL-35 spares regenerative angiogenesis at the late phase of HLI recovery after 14 dpi; *v)* Transcriptome analysis of ECs at 14 days post-HLI reveals a disturbed extracellular matrix re-organization in IL-35-injected mice; *vi)* IL-35 downregulates three reactive oxygen species (ROS) promoters and upregulates one ROS attenuator, which may functionally mediate IL-35 upregulation of anti-angiogenic extracellular matrix proteins in ECs; and *vii)* IL-35 inhibits human microvascular EC migration and tube formation *in vitro* mainly through upregulating anti-angiogenic extracellular matrix-remodeling proteins. In our study, we find that IL-35 inhibits ischemia/hypoxia-induced angiogenesis but spare regenerative angiogenesis. In summary, IL-35 regulates angiogenesis in a context-dependent manner, which is similar to the function of TGF-*β* and IL-10. This phenomenon may explain some unsatisfied outcome of CANTOS clinical trial.

The proper angiogenic process in which new blood vessels sprout from existing ones largely relies on timely regulation of ECM degradation and synthesis. Our results of GSEA of CD45^−^CD31^+^ ECs, isolated from IL-35-injected ischemic muscle at 14 dpi, reveal significant enrichment of gene sets regulating ECM remodeling, including collagen formation, MMP activation, and integrin *β*1 and *β*3 pathways. MMP and ADAM are the most-studied proteases in angiogenesis. Collectively, they can degrade all known ECM components. Our analysis reveals that three MMP genes (Mmp12, Mmp14, and Mmp19) are induced, and one endogenous inhibitor Timp4 is reduced in IL-35-treated ECs, which indicates an activated MMP pathway in IL-35-treated ECs at 14 dpi. MMPs are previously considered as pro-angiogenic mediators (88). However, it has also been well acknowledged that MMPs are also the principal proteases responsible for generating potent angiogenesis inhibitors such as angiostatin and endostatin (89, 90). Among them, MMP12 is potent in generating angiostatin (91) and endostatin (92) [COL18A1 is increased in IL-35-injected mouse ECs and IL-35-treated HMVECs (data not shown)]. Interestingly, there are also significant inductions of thrombospondin family genes (ECM glycoproteins, Thbs1, and Thbs2) in IL-35-injected mouse ECs. Thbs1 and Thbs2 have been well acknowledged as potent endogenous inhibitors of angiogenesis and tumor growth (93, 94). In addition, THBS1 protein level is elevated in IL-35-treated HMVECs. Collectively, abnormally activated ECM-remodeling genes at 14 dpi of HLI may explain the delayed blood flow recovery in IL-35-injected mice compared to PBS-injected control.

Ingenuity Pathway Analysis (IPA) core analysis of the 341 DEGs in the IL-35-injected mouse EC at 14 dpi predicts five top upstream regulators (**Figure 5C**). Among them, predicted downregulation of *α*-catenin could lead to 18 downstream molecules including collagen, and MMPs (**Figure 5D**). Though there is no significant change of *α*-catenin gene expression in IL-35-treated ECs in mice, it does not exclude the possibility of post-transcriptional regulation of *α*-catenin (95). In addition, hypoxia seems to induce the expression of *α*-catenin, while IL-35 inhibited the induction (**Figure 5E**). *α*-catenin, an actin-binding protein, is an element of the core complex in endothelial adherens junctions (AJs). The core complex is composed of vascular cadherin (VE)-cadherin (transmembrane protein), p120-catenin, *β*-catenin or plakoglobin (encoded by JUP gene), and *α*-catenin. Thus, we examined other components in our experiments, and the results showed that JUP (FC = 0.58) was significantly downregulated in the RNA-Seq data of mouse EC and VE-cadherin expression on EC was trending down as well (**Figures 5F, G**). This cadherin-based AJ regulates the intracellular actin-myosin network and is essential for vascular integrity (41). Studies have shown that *α*-catenin has a critical role in AJ development (96). Lack of VE-cadherin leads to increased vascular permeability and impaired endothelial barrier (42). Also, increased mRNA and protein level of plakoglobin has been associated with tightly confluent cells *in vitro* (97). These results suggest that endothelial AJ is impaired in the IL-35-injected mouse EC at 14 dpi. As we find in RNA-Seq result, integrin pathways are significantly enriched, which indicates an increase of focal adhesion (cell–ECM). Cell–ECM and cell–cell adhesions are highly integrated networks of protein interactions. Numerous studies have shown that integrins and cadherins share many downstream signaling molecules and can modulate each other in various ways (98, 99). A recent study shows that integrin can dissociate the VE-cadherin/catenin complex and disrupt vascular AJ (100). In addition, IL-35 inhibits HMVEC migration through including anti-angiogenic proteins of pigment epithelium-derived factor (PEDF), Maspin, and thrombospondin-1 (THBS-1). All three proteins have been shown to inhibit EC migration *in vitro*. PEDF is a potent inhibitor of EC migration under many angiogenic inducers, including PDGF, VEGF, and IL-8 (74). Maspin, a tumor suppressor, has been shown to inhibit HUVEC migration under FGF2 stimulation through an integrin *β*1 signaling pathway (75). This study shows that Maspin increases focal adhesion stability through activation of integrin-linked kinases and retards EC migration. Besides, THBS-1, an endogenous inhibitor of angiogenesis which is also induced in IL-35-injected mouse EC *in vivo*, inhibits HUVEC migration in an integrin *β*1-dependent manner (76). It shows that the type-1 repeat (TSR) domain in THBS-1 can bind to *β*1 integrins and inhibit EC migration. Thus, we propose that IL-35-enhanced integrin-mediated cell–ECM adhesion may lead to the disassembling (VE-cadherin)–plakoglobin–(*α*-catenin) complex, which impairs vascular AJ and EC migration. However, further experiment should be set up to test this hypothesis.

**Figure 8:**
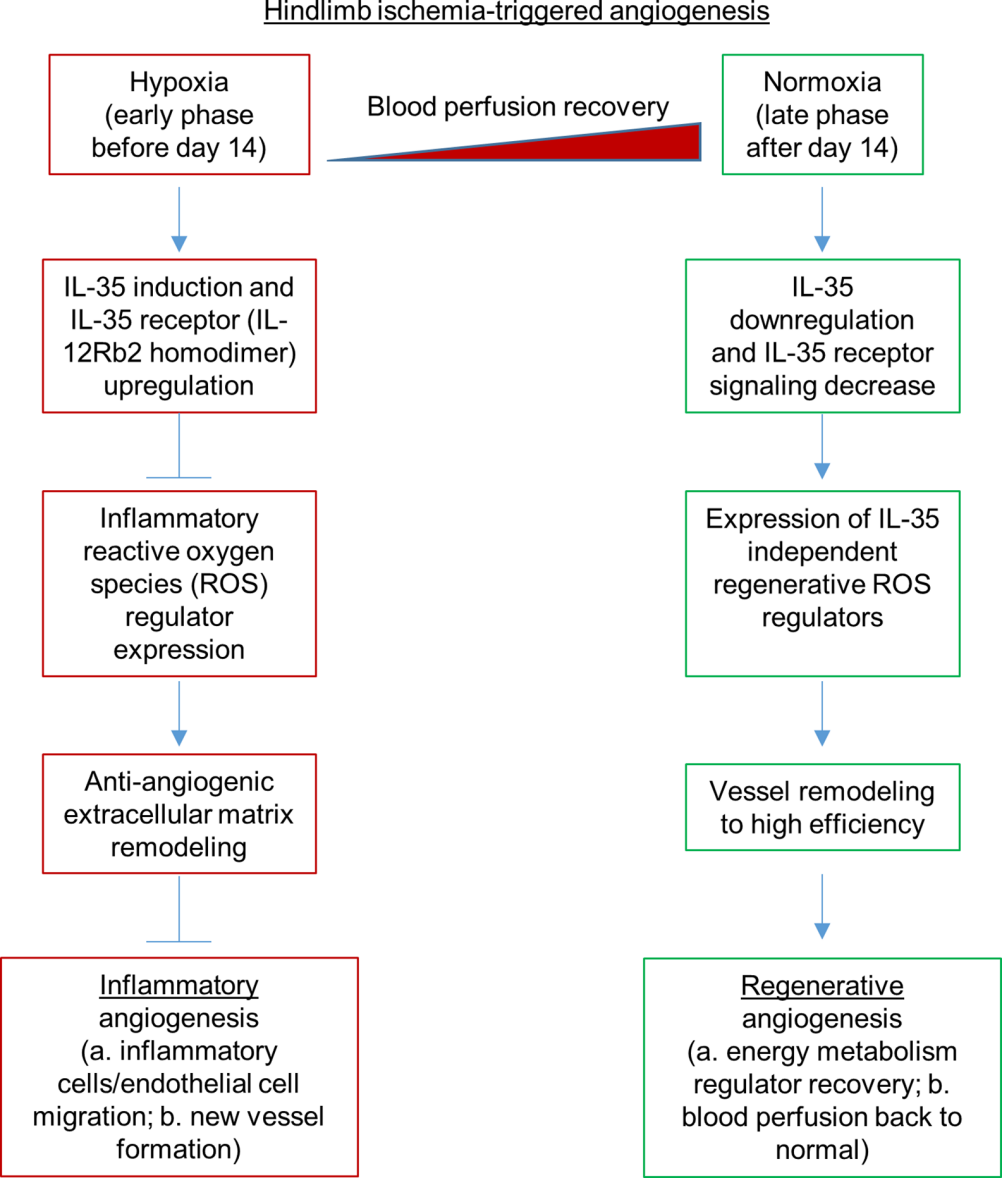
A new working model: Hypoxia-induced anti-inflammatory cytokine IL-35 inhibits hindlimb ischemia-triggered inflammatory angiogenesis at early phase, but spare regenerative angiogenesis at late phase.

Based on our and others’ findings, we propose a novel working model (**Figure 8**): *first*, HLI triggered angiogenesis can be classified into two phases: 1) we define early phase as before the day 14 after HLI surgery based on the data presented in **Figure 2B**. We observed a significant improvement of blood supply in ApoE−/−/IL-12Rb2−/− mice in the day 14 after surgery comparing to that at the day 7 after surgery but no significant differences between ApoE KO and ApoE−/−-/IL-12Rb2−/− mice. 2) We observed that in **Figure 3B**, two curves converged at day 28 after surgery, which indicates that IL-35-treated group has at least two phases of the effect on the hypoxia phase and recovery phase after HLI. HLI-created hypoxia triggers inflammatory angiogenesis at early phase 1 to 14 dpi as judged by upregulating 18 chemokines and receptors, 23 cytokines, and receptors. We previously reported a list of 14 proangiogenic cytokines and chemokines (38). These proangiogenic cytokines/chemokines were upregulated at the early phase of HLI (unpublished). *Second*, HLI induces upregulation of anti-inflammatory cytokine IL-35, which inhibits inflammatory angiogenesis by downregulating ROS regulators DDIT4 and XDH and upregulating anti-angiogenic ECM mechanisms up to 14 dpi. *Third*, after 14 dpi hypoxia gradually changes to normoxia at the late phase and regenerative angiogenesis as judged by upregulating energy metabolism regulators, vessel remodeling to high efficiency, and blood perfusion restored back to normal; *fourth*, out of 165 ROS regulators in the GSEA database, the expressions of 116 ROS regulators are significantly modulated in HLI, which can be classified into the early phase inflammatory angiogenesis ROS regulators (25 regulators upregulated at 3 dpi) and late phase regenerative angiogenesis ROS regulators (56 regulators upregulated at 7 dpi) (**Supplementary Figure 5**). IL-35 spares regenerative angiogenesis after 14 dpi in HLI by anti-ROS regulator Fbln5, which are well correlated with recent reports that IL-35 is protective in myocardial infarction-induced injury (85, 101). Of note, the stronger protection of IL-35 in the cardiac muscle than in the skeletal muscle may be due to tissue specificity. In addition, differences between the IL-35 roles in angiogenesis in HLI and myocardial infarction may also result from potential functions of cardiokines (86) and myokines (87). These findings provide a novel insight on the future therapeutic potential of IL-35 in suppressing ischemia/inflammation-triggered inflammatory angiogenesis at the early phase but sparing regenerative angiogenesis at the late phase, which is similar to our recent report that IL-35 inhibits human aortic endothelial cell activation triggered by proatherogenic lipids but spare trained immunity pathway (8).

## Data Availability Statement

The datasets presented in this study can be found in online repositories. The names of the repository/repositories and accession number(s) can be found in the article/**Supplementary Material**.

## Ethics Statement

The animal study was reviewed and approved by Temple University.

## Author Contributions

HF carried out the experiments, data gathering and data analysis and prepared tables and figures. YSu, YSh, JSa, RC, LL, CD, CJ, KX, YL, XL, SM, EX, JT, NJ, DY, YZ, KB, JY, TR, WH, NS, JSu, XQ, XJ, and HW aided with analysis of the data. XY supervised the experimental design, data analysis, and manuscript writing. All authors contributed to the article and approved the submitted version.

## Funding

This work was funded by the National Institutes of Health Grants to XY and HW.

## Conflict of Interest

The authors declare that the research was conducted in the absence of any commercial or financial relationships that could be construed as a potential conflict of interest.

## Supplementary Material

Supplementary Figure 1 IL-35 is induced by hypoxia treatment in human microvascular endothelial cells (HMVECs) and its receptor subunit IL-12RB2, but not IL-6ST, is increased by IL-35 treatment. **(A)** Gene expressions of IL-35 subunits in GSE3313 dataset. Whole muscle RNA was collected from male mice, and expression levels were compared to that of pre-HLI surgery. **(B)** Experimental schematics of hypoxia treatment on HMVECs. After HMVECs were 80% confluent, the hypoxia group was incubated in a hypoxia chamber (BioSpherix, P110) at 0.2% O_2_ for indicated hours. Western blot of hypoxia inducible factor 1 subunit alpha (HIF1A) protein level was significantly increased after 6-h hypoxia treatment. **(C)** Normalized mRNA expression levels of IL-35 and its receptor subunits after 2, 6, and 24 h of hypoxia treatment. Data are presented as mean ± SEM with individual points plotted. *p < 0.05; **p < 0.01; ***p < 0.001; ****p < 0.0001.

Supplementary Figure 2 IL-35-IL12RB2 signaling inhibits hindlimb ischemia-induced angiogenesis specially in male mice; male outperforms female regardless to the treatment or genotype. **(A–F)** Blood perfusion ratio in HLI model. Hindlimb blood flow was measured on day post-ischemia (dpi) 0, 3, 7, 14, 21, and 28 using a laser Doppler blood flow analyzer. Blood flow was quantitatively assessed by the ratio of mean flow signals of the ischemic to the non-ischemic plantar. Data are presented as mean ± SEM with individual points plotted. *p < 0.05; **p < 0.01; ***p < 0.001; ****p < 0.0001.

Supplementary Figure 3 IL-35 inhibits FGF2-induced angiogenesis in Matrigel plug assay. **(A)** Schematics of Matrigel plug assay and findings. Blue lines represented the extracellular matrix, red lines represented blood vessels. **(B)** Representative images of the Matrigel plugs (~1.5 cm of diameter attached to the skin) at 11 days after injection. Images were taken under dissecting microscope. Black arrows indicated the new blood vessels grown in the Matrigel plug. **(C)** Representative images of Matrigel plug stained with anti-CD31 (green), anti-neuron-glial (NG2, red, a pericyte marker), anti-F4/80 (magenta), and DAPI (blue). White dashed lines contour the attached skin at below. **(D)** Statistics of cell count and the percentage of indicated populations. n = 5. Statistics were based on 45 randomly selected field of views. Scale bar sized are as indicated in the pictures. All results were collected from male mice. Data are presented as mean ± SEM with individual points plotted. *p < 0.05; **p < 0.01; ***p < 0.001; ****p < 0.0001.

Supplementary Figure 4 47 collagen genes are analyzed in RNA-seq data.

Supplementary Figure 5 The expressions of 116 out of 165 reactive oxygen species regulators in the GSEA database were significantly changed on the day 1, day 7, and day 14 after hindlimb ischemia (HLI) in the wild-type male mouse datasets in the GSE3313 datasets in the NIH Geo Datasets database. The significantly changed genes were listed in this table and heatmap on the left panel (As shown in **Figure 6B**, DDIT4 and XDH shown in blue fonts were suppressed by IL-35, and FBLN5 shown in red fonts was increased by IL35. TNF was not significantly changed in the time course).

Supplementary Figure 6 IL-35 induces anti-angiogenic protein of pigment epithelium-derived factor (PEDF) in human microvascular endothelial cells (HMVECs). **(A, B)** Immunoblots and statistics of PEDF expression levels. After starvation with 0.1% FBS overnight, HMVECs were treated with 40 ng/ml of IL-35 for indicated time. **(C, D)** Immunoblots and statistics of PEDF expression levels. After starvation with 0.1% FBS overnight, HMVECs were treated under normoxia, or hypoxia (0.2% O_2_) with or without 40 ng/ml of IL-35 for 12 h. Data are presented as mean ± SEM. *p < 0.05; **p < 0.01; ***p < 0.001; ****p < 0.0001.
