# Supplementary figures and images for: Interleukin 35 Delays Hindlimb Ischemia-Induced Angiogenesis Through Regulating ROS-Extracellular Matrix but Spares Later Regenerative Angiogenesis
Source: Front Immunol. 2020 Oct 14;11:595813. doi: 10.3389/fimmu.2020.595813 (PMC7591706; doi:10.3389/fimmu.2020.595813)

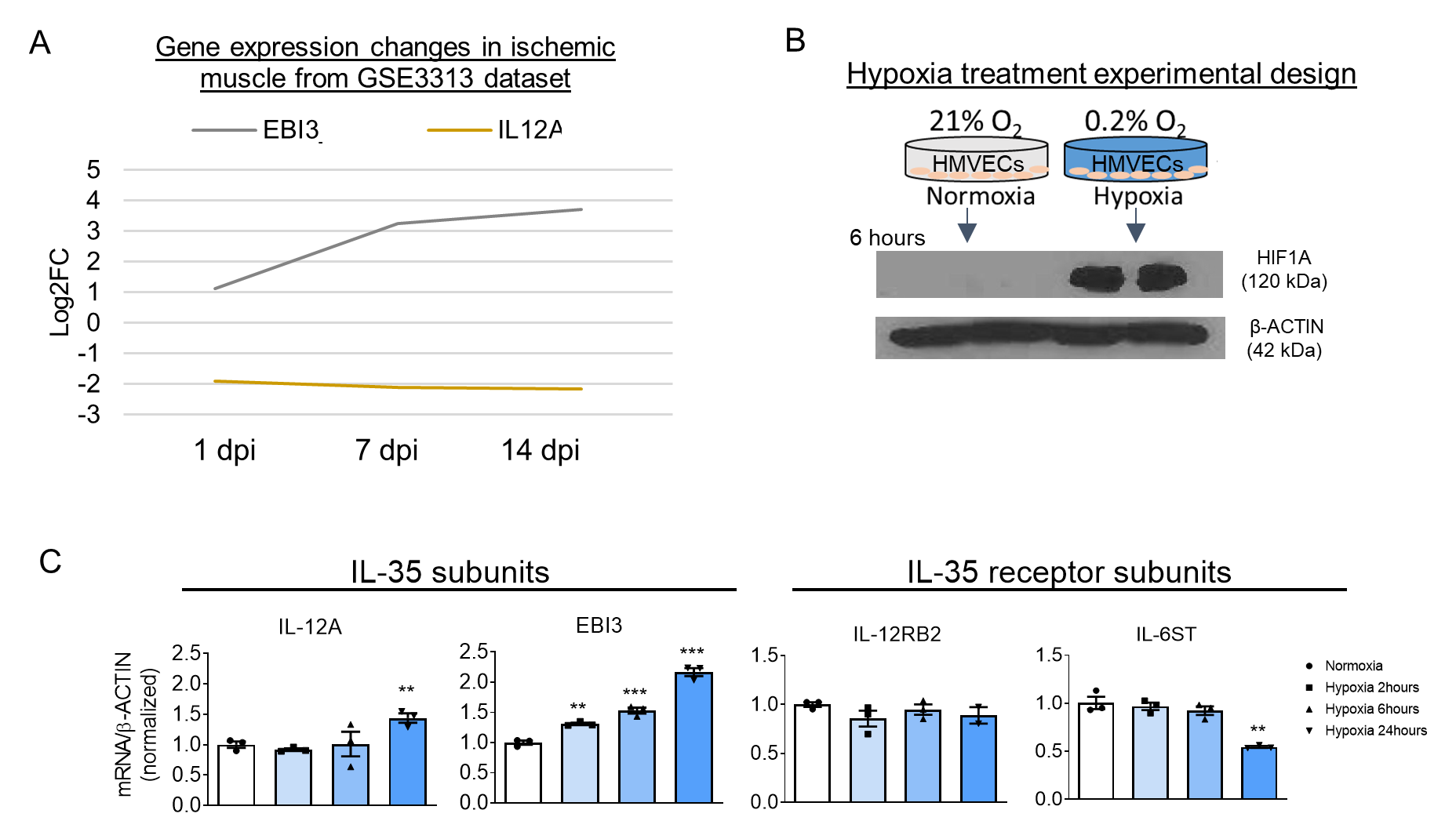

Supplement: Supplementary Figure 1 — IL-35 is induced by hypoxia treatment in human microvascular endothelial cells (HMVECs) and its receptor subunit IL-12RB2, but not IL-6ST, is increased by IL-35 treatment. (A) Gene expressions of IL-35 subunits in GSE3313 dataset. Whole muscle RNA was collected from male mice, and expression levels were compared to that of pre-HLI surgery. (B) Experimental schematics of hypoxia treatment on HMVECs. After HMVECs were 80% confluent, the hypoxia group was incubated in a hypoxia chamber (BioSpherix, P110) at 0.2% O2 for indicated hours. Western blot of hypoxia inducible factor 1 subunit alpha (HIF1A) protein level was significantly increased after 6-h hypoxia treatment. (C) Normalized mRNA expression levels of IL-35 and its receptor subunits after 2, 6, and 24 h of hypoxia treatment. Data are presented as mean ± SEM with individual points plotted. *p < 0.05; **p < 0.01; ***p < 0.001; ****p < 0.0001. [file Image_1.tif]

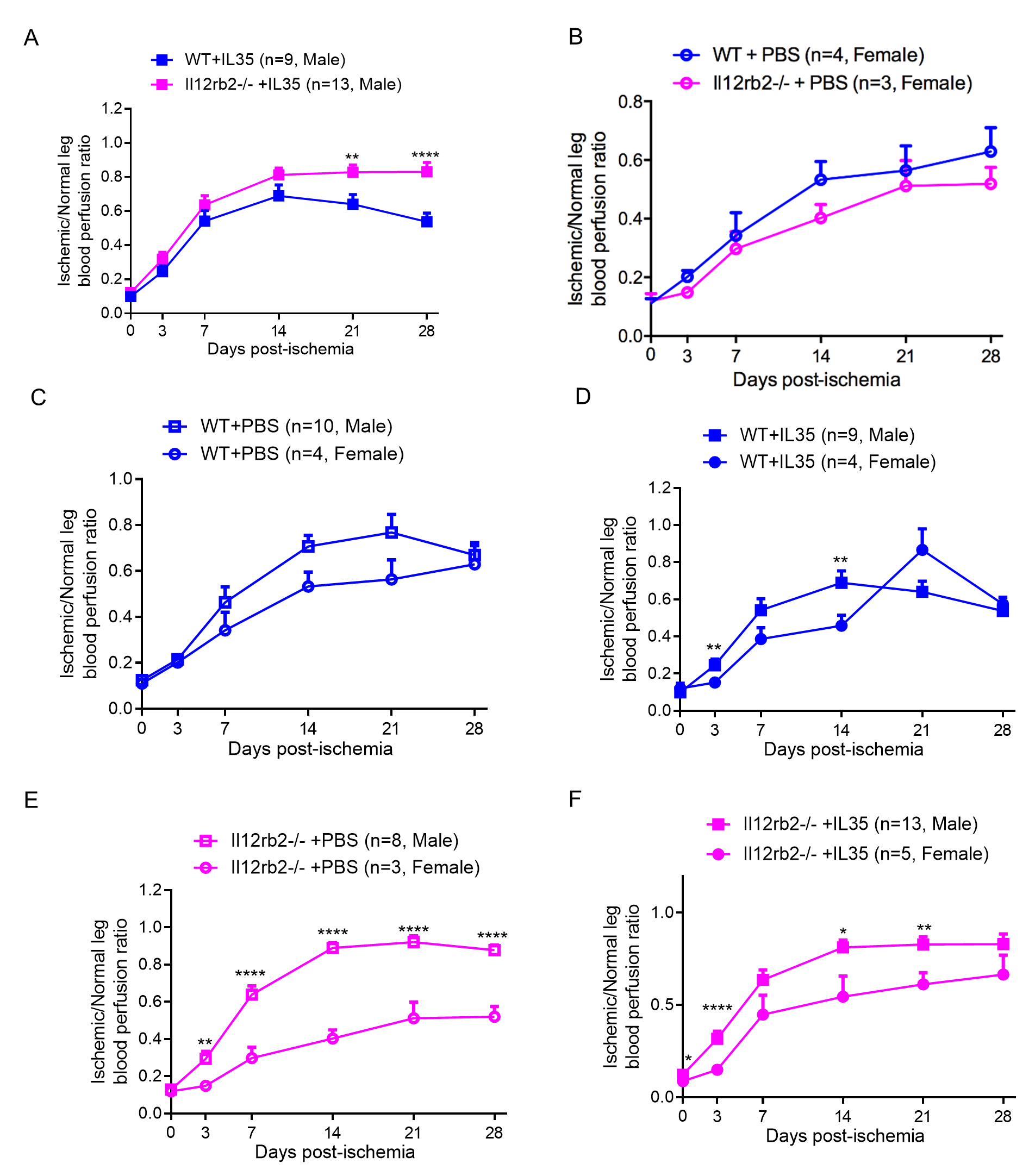

Supplement: Supplementary Figure 2 — IL-35-IL12RB2 signaling inhibits hindlimb ischemia-induced angiogenesis specially in male mice; male outperforms female regardless to the treatment or genotype. (A–F) Blood perfusion ratio in HLI model. Hindlimb blood flow was measured on day post-ischemia (dpi) 0, 3, 7, 14, 21, and 28 using a laser Doppler blood flow analyzer. Blood flow was quantitatively assessed by the ratio of mean flow signals of the ischemic to the non-ischemic plantar. Data are presented as mean ± SEM with individual points plotted. *p < 0.05; **p < 0.01; ***p < 0.001; ****p < 0.0001. [file Image_2.tif]

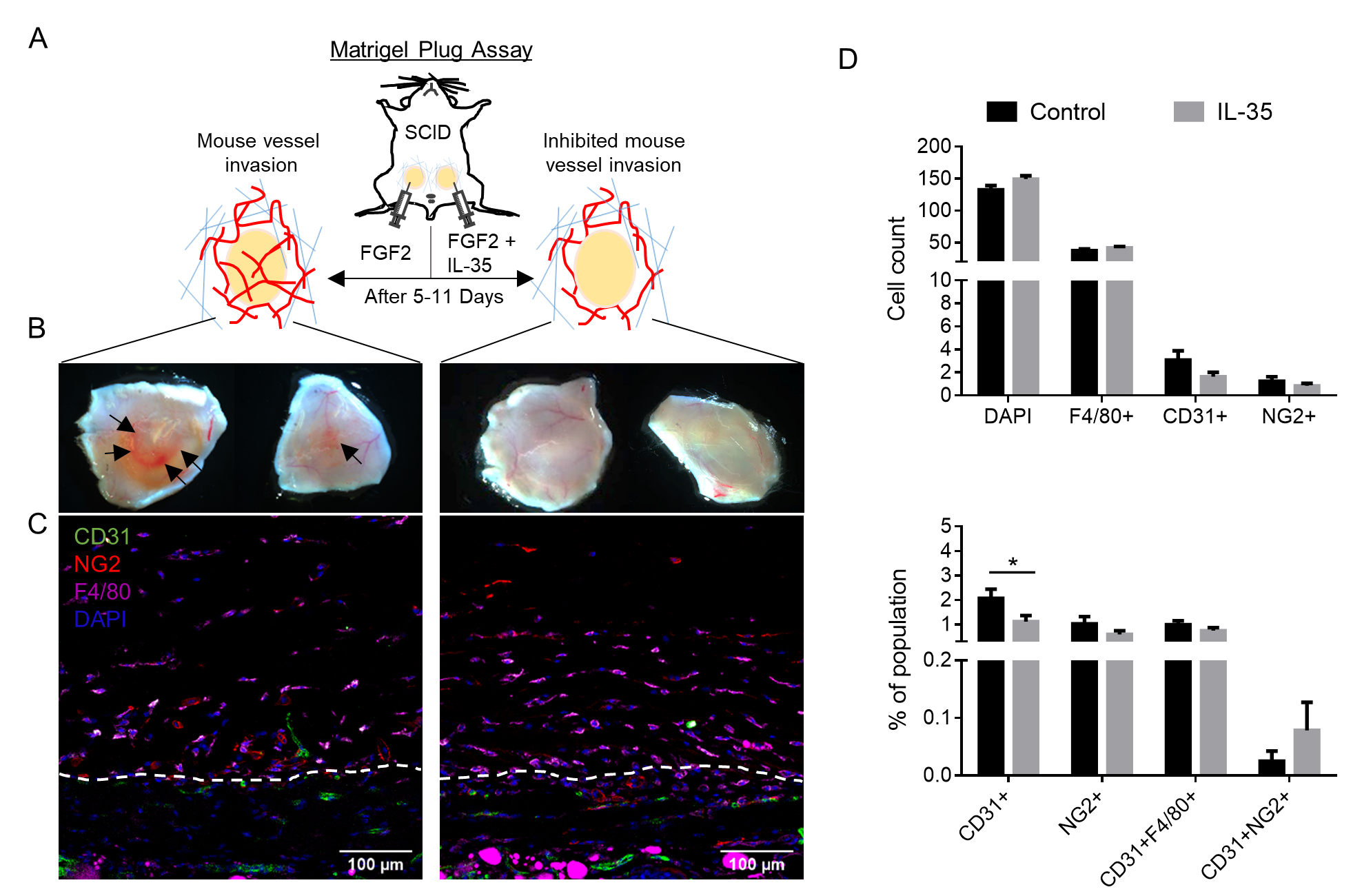

Supplement: Supplementary Figure 3 — IL-35 inhibits FGF2-induced angiogenesis in Matrigel plug assay. (A) Schematics of Matrigel plug assay and findings. Blue lines represented the extracellular matrix, red lines represented blood vessels. (B) Representative images of the Matrigel plugs (~1.5 cm of diameter attached to the skin) at 11 days after injection. Images were taken under dissecting microscope. Black arrows indicated the new blood vessels grown in the Matrigel plug. (C) Representative images of Matrigel plug stained with anti-CD31 (green), anti-neuron-glial (NG2, red, a pericyte marker), anti-F4/80 (magenta), and DAPI (blue). White dashed lines contour the attached skin at below. (D) Statistics of cell count and the percentage of indicated populations. n = 5. Statistics were based on 45 randomly selected field of views. Scale bar sized are as indicated in the pictures. All results were collected from male mice. Data are presented as mean ± SEM with individual points plotted. *p < 0.05; **p < 0.01; ***p < 0.001; ****p < 0.0001. [file Image_3.tif]

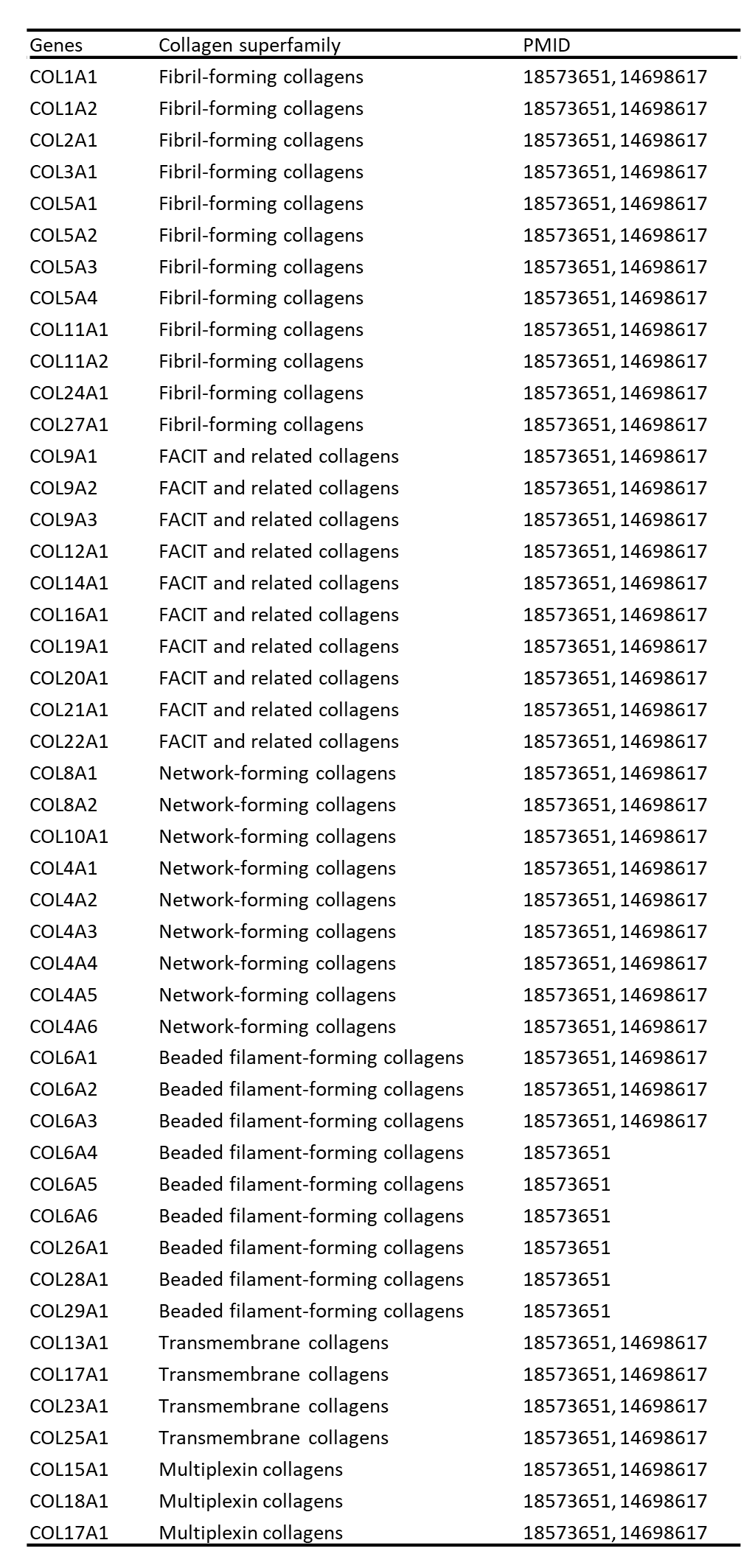

Supplement: Supplementary Figure 4 — 47 collagen genes are analyzed in RNA-seq data. [file Image_4.tif]

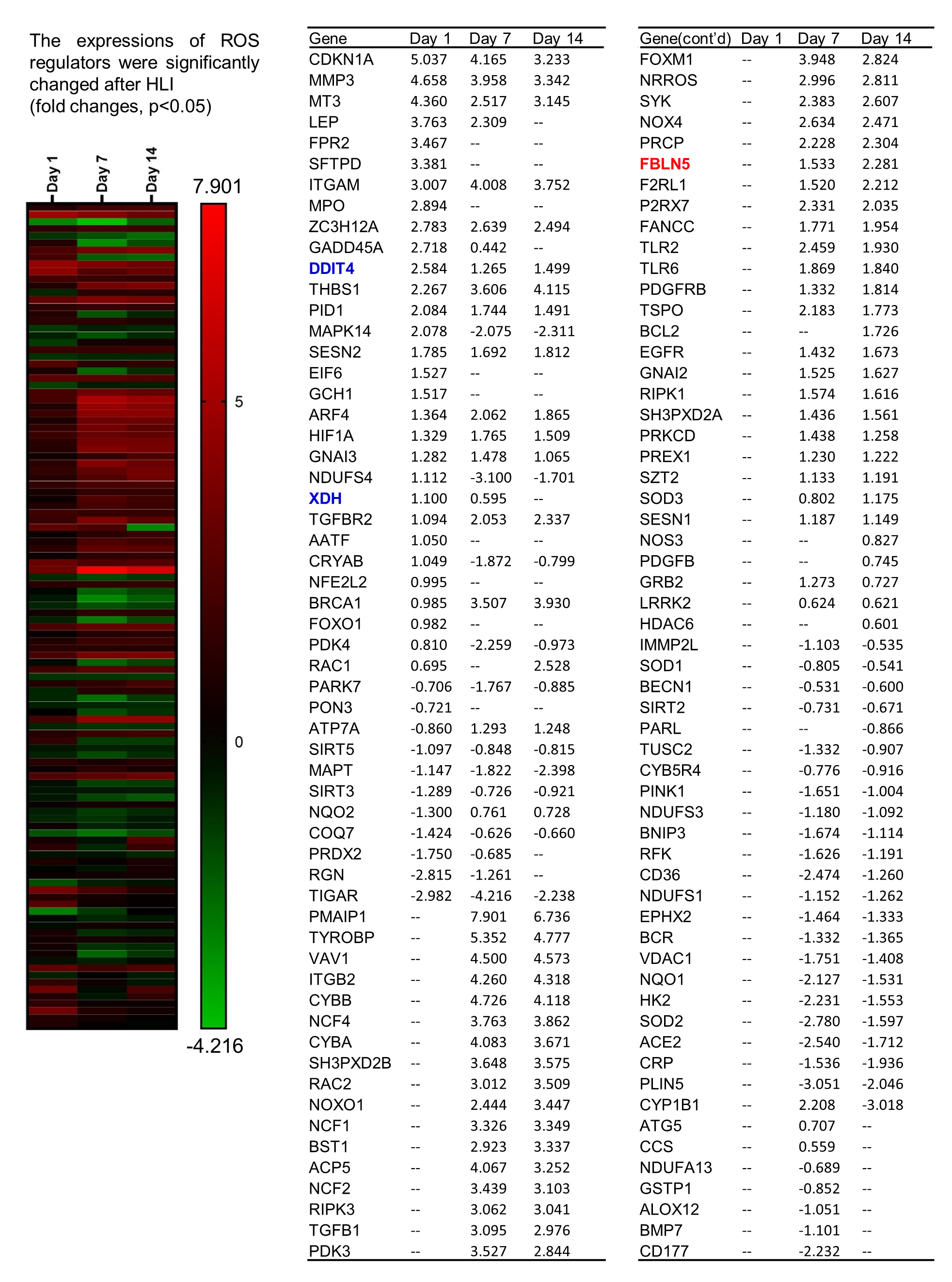

Supplement: Supplementary Figure 5 — The expressions of 116 out of 165 reactive oxygen species regulators in the GSEA database were significantly changed on the day 1, day 7, and day 14 after hindlimb ischemia (HLI) in the wild-type male mouse datasets in the GSE3313 datasets in the NIH Geo Datasets database. The significantly changed genes were listed in this table and heatmap on the left panel (As shown in Figure 6B, DDIT4 and XDH shown in blue fonts were suppressed by IL-35, and FBLN5 shown in red fonts was increased by IL35. TNF was not significantly changed in the time course). [file Image_5.tif]

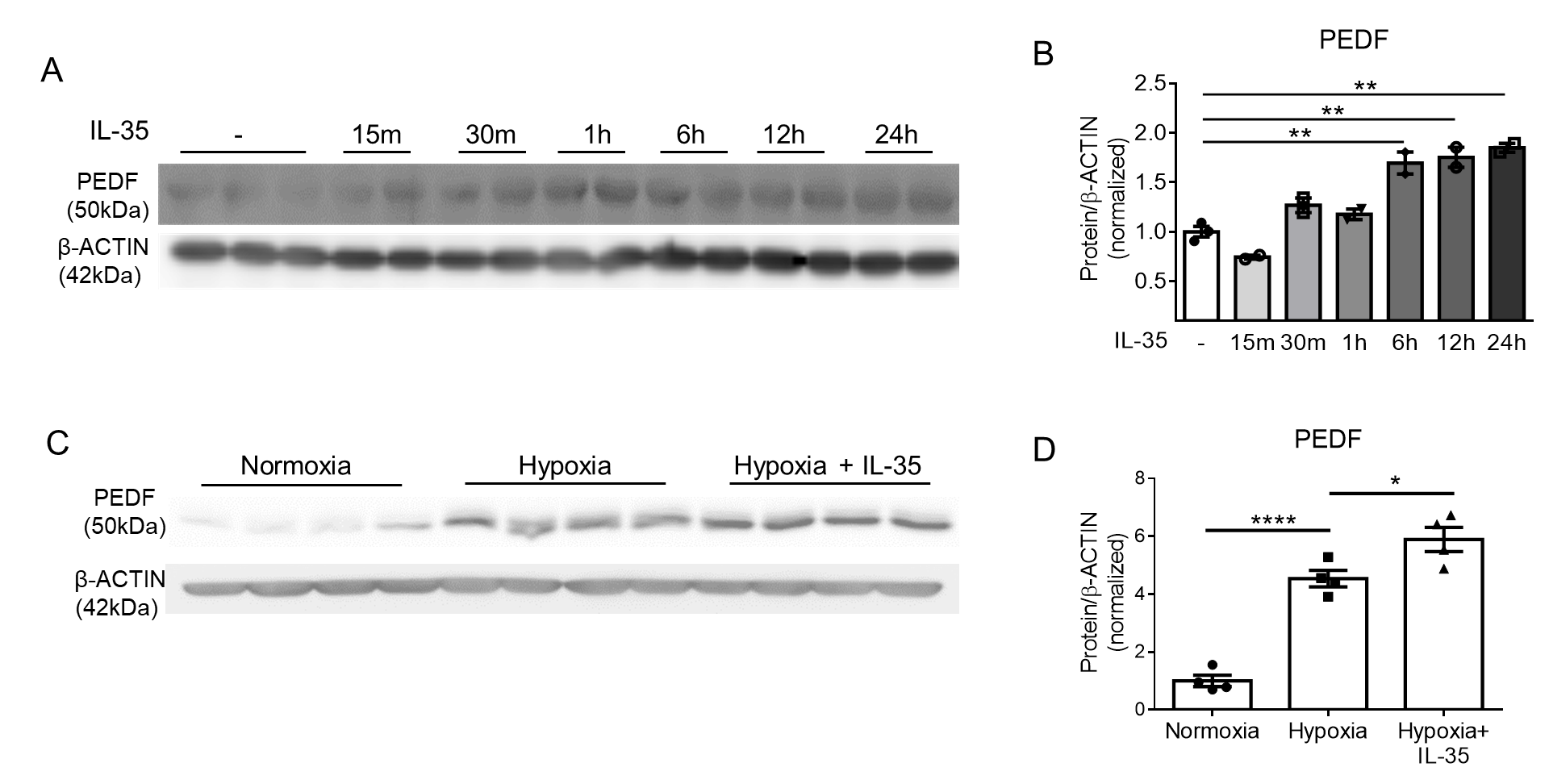

Supplement: Supplementary Figure 6 — IL-35 induces anti-angiogenic protein of pigment epithelium-derived factor (PEDF) in human microvascular endothelial cells (HMVECs). (A, B) Immunoblots and statistics of PEDF expression levels. After starvation with 0.1% FBS overnight, HMVECs were treated with 40 ng/ml of IL-35 for indicated time. (C, D) Immunoblots and statistics of PEDF expression levels. After starvation with 0.1% FBS overnight, HMVECs were treated under normoxia, or hypoxia (0.2% O2) with or without 40 ng/ml of IL-35 for 12 h. Data are presented as mean ± SEM. *p < 0.05; **p < 0.01; ***p < 0.001; ****p < 0.0001. [file Image_6.tif]
